# On indirect negative inotropic effects of muscarine in the isolated human atrium

**DOI:** 10.1007/s00210-026-05666-2

**Published:** 2026-07-14

**Authors:** Joachim Neumann, Britt Hofmann, Katarina Hadova, Jan Klimas, Ulrich Gergs

**Affiliations:** 1 Institute for Pharmacology and Toxicology, Medical Faculty, Martin Luther University Halle-Wittenberg, Magdeburger Straße 4, 06097 Halle (Saale), Germany; 2 Cardiac Surgery, Mid-German Heart Centre, Martin Luther University Halle-Wittenberg, Ernst-Grube-Straße 40, 06097 Halle, Germany; 3 Department of Pharmacology and Toxicology, Faculty of Pharmacy, Comenius University, Odbojárov 10, SK 83232 Bratislava 3, Slovak Republic

**Keywords:** Carbachol, Muscarine, Acetylcholine, Isoprenaline, Pilocarpine, Semaglutide, Cilostamide, Human atrium

## Abstract

Acetylcholine, the physiological agonist at all muscarinic receptors, alone (“direct effect”) or after pre-stimulation with isoprenaline (“indirect effect”), is known to reduce the force of contraction (FOC) in human atrial preparations (HAP). We hypothesized that muscarine might act similar to acetylcholine. During open heart surgery for severe coronary heart disease, HAP were obtained. Using digital polymerase chain reaction, we noted that the messenger ribonucleic acid (mRNA) of the M_1_-, M_2_-, and M_3_-muscarinic receptors in HAP contributed to 0.09%, 96.34%, and 3.57%, respectively, of the total mRNA expression of the combined mRNA expression of the sum of the M_1_-, M_2_-, and M_3_-muscarinic receptors. The HAP were mounted in organ baths, electrically stimulated (1 Hz) and FOC was measured under isometric conditions. When given alone, muscarine transiently reduced FOC. After application of 1 µM isoprenaline (to stimulate β-adrenoceptors) or 1 µM serotonin (to stimulate 5-HT_4_-serotonin receptors) or 1 µM histamine (to stimulate H_2_-histamine receptors) or 10 µM fenoldopam (to stimulate D_1_-dopamine receptors) or 100 nM semaglutide (to stimulate glucagon-like-peptide-1 receptors) or 100 nM gastric inhibitory peptide (to stimulate the gastric inhibitory peptide receptors) additionally applied 1 µM muscarine reduced FOC. Interestingly, after 1 µM forskolin (to stimulate adenylyl cyclase independent of receptor involvement), 1 µM cilostamide (to inhibit cAMP degradation) or 100 µM dibutyryl-cAMP (to directly stimulate the cAMP-dependent protein kinase), additionally applied 1 µM muscarine also reduced FOC. We conclude that muscarine in the presence of several cAMP-increasing agents or a cAMP-dependent protein kinase activating agent exerts negative inotropic effects in HAP. These contractile responses might underlie negative inotropic effects in muscarine intoxications in the clinic.

**Supplementary Information:**

The online version contains supplementary material available at https://doi.org/10.1007/s00210-026-05666-2.

## Introduction

We and others have studied the contractile effects of the M-cholinoceptor agonists carbachol and acetylcholine in an isolated cardiac preparation from experimental animals and from humans (e.g., Jacob et al. 1989; Böhm et al. 1994; Du et al. 1994; Schwarz et al. 2023). Usually in mammals, acetylcholine per se has a (small, “direct effect”) negative inotropic effect in atria which was augmented (large, “indirect effect”) after β-adrenergic stimulation but not after, e.g., α-adrenergic stimulation (Levy (1971) accentuated antagonism).

In the present work, we were interested in the contractile effects of the unselective M-cholinoceptor agonist muscarine on the force of contraction in isolated electrically stimulated muscle preparations from the human right atrium (HAP). Muscarine is not used as a therapeutic drug but sometimes poses a problem in clinical toxicology. In such intoxications, muscarine can enter the human body when eating certain fungi (Schmiedeberg and Koppe 1869; Bowden et al. 1965; Lurie et al. 2009; Graeme 2014; Patocka et al. 2021). Muscarine intoxications have caused death in humans (e.g., Nicholson and Korman 1997). Muscarine is a quaternary amine, and hence, it is thought not to easily pass the blood–brain barrier, though toxic effects on the central nervous system seem to occur in rare cases (Goodman and Gilman 2023). Typical signs of intoxication with muscarine are sweating, salivation, abdominal pain, vomiting and watery diarrhea, in other words, expected signs of parasympathetic stimulation (e.g., Nicholson and Korman 1997). All five M-cholinoceptors have been detected in the human heart on the messenger ribonucleotide (mRNA) level (Wang et al. 2001). The M_2_-cholinoceptor mediates in all likelihood the negative inotropic effect of M-cholinoceptor agonists like acetylcholine or carbachol in HAP (Du et al. 1995). However, stimulation of M_1_-cholinoceptors (in human atrium) or M_3_-cholinoceptors (in mouse atrium, in canine Purkinje fibers) may even exert a positive inotropic effect (Du et al. 1995; Dhein et al. 2001; Tsuchida et al. 2013). The role of M_4_-cholinoceptors and M_5_-cholinoceptors for cardiac inotropy is currently not understood and will therefore not be considered further (Dhein et al. 2001).

Both acetylcholine and carbachol alone or in the presence of isoprenaline have been used before to stimulate muscarinic receptors in HAP (e.g., Löffelholz and Pappano 1985, Jacob et al. 1989; Böhm et al. 1994; Schwarz et al. 2023). However, to the best of our knowledge, no report on the inotropic effects in HAP of muscarine alone or in the presence of β-adrenergic stimulation can be found.

**Fig. 1:**
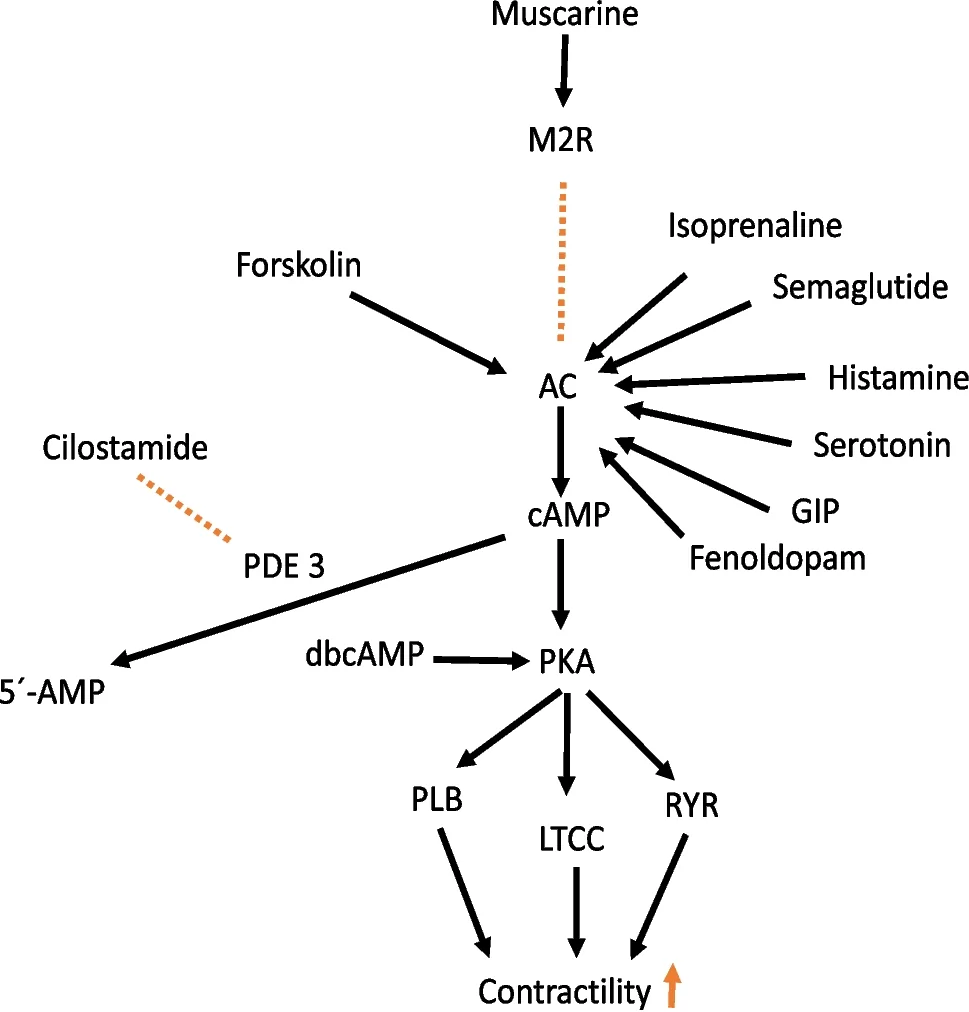
Putative mechanism(s) of action of muscarine in the human atrium. Muscarine stimulates the M_2_-muscarinic receptor (M2R). This may lead to an inhibition (brown broken line) of the adenylyl cyclase (AC). Isoprenaline stimulates via β-adrenoceptors the activity of AC. In a similar fashion, fenoldopam, semaglutide, serotonin, gastrin stimulatory peptide (GIP) and histamine stimulate D_1_-dopamine receptors or glucagon-like protein-1 receptors or serotonin 4 receptors or GIP receptors or histamine 2 receptors, respectively. Thus, they increase the activity of AC, cAMP and force. Forskolin directly stimulates the AC. Dibutyryl-cAMP (db-cAMP) directly stimulates the cAMP-dependent protein kinase (PKA). PKA phosphorylates regulatory proteins like phospholamban (PLB), the L-type Calcium ion channel (LTCC) and the ryanodine receptor (RYR). These phosphorylations increase Ca2 + and therefore lead to an increase in contractility (vertical red arrow) in the isolated human atrium. AC forms cAMP that is degraded in the human heart mainly by phosphodiesterase 3 (PDE 3) which is inhibited (brown broken line) by cilostamide

Others noted that acetylcholine alone or carbachol alone exerted transient negative inotropic effects that peaked after 2 min (at latest) and then a positive inotropic effect followed (mouse atrium: Nishimaru et al. 2000; Tanaka et al. 2001; Hara et al. 2009; human atrium, e.g., Jacob et al. 1989). Carbachol or acetylcholine attenuates the positive inotropic effects of isoprenaline (“indirect effect”). This is well studied in the mammalian ventricle. It is important that such “indirect” anti-adrenergic effects of carbachol also occur in isolated muscle strips from the human atrium (e.g., Genovese et al. 1988). We asked whether muscarine exerts not only a direct negative inotropic effect but also an indirect negative inotropic effect, i.e., in the presence of isoprenaline. We were further interested whether the positive inotropic effect of other drugs that also activate the cAMP formation via stimulatory GTP-binding proteins could be diminished by muscarine (Fig. 1). More specifically, one question was whether the indirect effect of muscarine was dependent on the receptor that raises cAMP in the human atrium. All receptors studied here (Fig. 1) are known to exert a positive inotropic effect in HAP. In principle, any negative inotropic effect of M_2_-muscarinic receptor stimulation may result from inhibition of adenylyl cyclase activity, activation of protein phosphatases and/or interaction with ion channels (mainly potassium ion channels or L-type calcium ion channels) in the sarcolemma (discussed in Dhein et al. 2001; Schwarz et al. 2023). Therefore, we were interested in the signal transduction of muscarine, from the receptor to the generation of force (Fig. 1). We have presented evidence before that the action of carbachol might be independent, at least in part, from an inhibition of the cAMP formation, but that other mechanisms like phosphatase activation might be involved (Schwarz et al. 2023). Therefore, we used pharmacological tools that we had employed before to delineate the mechanism(s) of action of carbachol or adenosine in HAP (Schwarz et al. 2023, 2024, 2025). Specifically, we interrogated whether the negative inotropic effect of muscarine is also present if we stimulated the generation of cAMP independent of a receptor (with forskolin), by inhibiting the degradation of cAMP (with cilostamide), or by direct activation of cardiac cAMP-dependent protein kinases (with dibutyryl-cAMP, Fig. 1). For comparison with muscarine, we tested three well-established M-cholinoceptor agonists, notably pilocarpine, carbachol and acetylcholine itself.

Thus, we tested the following main hypotheses:

Firstly, muscarine exerts indirect negative inotropic effects when we stimulate receptors that are known to increase cAMP in HAP.

Secondly, these indirect negative inotropic effects of muscarine in HAP may be independent of an inhibition of the activity of the adenylyl cyclase.

## Materials and methods

### Contractile studies on human preparations

The bathing solution of the organ baths contained 119.8 mM NaCl, 5.4 mM KCl, 1.8 mM CaCl_2_, 1.05 mM MgCl_2_, 0.42 mM NaH_2_PO_4_, 22.6 mM NaHCO_3_, 0.05 mM Na_2_EDTA, 0.28 mM ascorbic acid, and 5.05 mM glucose. The solution was continuously gassed with 95% O_2_ and 5% CO_2_ and maintained at 37 °C and pH 7.4 (Gergs et al. 2017). Human atrial preparations were stimulated with square waves for 5 ms duration with a voltage 10% higher than the threshold to initiate contractions. Under isometric conditions, we fed the signals to a bridge amplifier and then the signals went into a commercial personal computer.

The drug application was as follows. After equilibration was reached, M-cholinoceptor agonists were cumulatively applied or non-cumulatively applied. Then, we performed a washout. Thereafter, we applied 1 µM isoprenaline for 5 min or the indicated other positive inotropic drugs (Fig. 1B) and then gave again cumulatively or non-cumulatively the M-cholinoceptor agonists.

The cardiac right atrial muscle samples (HAP) were obtained from 12 male patients and four female patients, aged 56 to 82 years. Cardiac morbidities included severe coronary heart disease (two or three vessel disease), arterial hypertension, chronic ischemic heart disease with heart failure, peripheral arterial occlusion and atrial fibrillation. Additional morbidities were bladder cancer, insulin-dependent diabetes mellitus, obesity, spondyloarthritis, hyperlipidemia, gonarthrosis, epilepsy, pneumonia and herpes zoster. Cardiac drug therapy comprised acetyl salicylic acid, apixaban (and other anticoagulants), furosemide (and other diuretics), eplerenon, amlodipine, bisoprolol (and other β-adrenoceptor antagonists), dapaglifloxin, candesartan (and other angiotensin-2 receptor antagonists), ramipril (and other angiotensin-converting enzyme inhibitors) and statins. Our methods used for atrial contraction studies in human samples have been previously published and were not altered in this study (Gergs et al. 2009, 2017). Patients gave written informed consent to participate in this study. We had a permit from the local ethical committee.

Human right atrial tissue samples that were not required for contraction studies were frozen in liquid nitrogen and kept at − 80 °C in a freezer. These samples were homogenized under liquid nitrogen, and total RNA was isolated using acid phenol–guanidinium thiocyanate–chloroform extraction (TRI Reagent®, Sigma-Aldrich) in accordance with the manufacturer’s protocol. RNA was precipitated from the aqueous phase with isopropanol and subsequently washed twice with 80% ethanol. The integrity and quality of the isolated RNA were assessed by electrophoresis on a 2% agarose gel. Reverse transcription was performed using intact RNA samples with the High-Capacity cDNA Reverse Transcription Kit supplemented with RNase inhibitors (Applied Biosystems, Grand Island, NY, USA), following the manufacturer’s instructions. Quantification of mRNA transcripts encoding M_1_-muscarinic receptors, M_2_-muscarinic receptors and M_3_ -muscarinic receptors in each sample was carried out using digital polymerase chain reaction (dPCR) on the QuantStudio™ Absolute Q™ Digital PCR System (Applied Biosystems, Grand Island, NY, USA). Reactions were prepared using the Absolute Q™ DNA Digital PCR Master Mix (Applied Biosystems, Grand Island, NY, USA). Gene-specific expression was detected using predesigned TaqMan® Gene Expression Assays (Applied Biosystems, Grand Island, NY, USA): assay ID Hs00912795_m1 for M_1_-muscarinic receptors, Hs00265208_s1 for M_2_-muscarinic receptors, and Hs01374665_m1 for M_3_-muscarinic receptors. Absolute quantification results, expressed as copies per microliter (cp/µL), were normalized to the expression level of the housekeeping gene HPRT1 (assay ID: Hs02800695_m1). dPCR reactions were performed on QuantStudio™ Absolute Q™ MAP16 Digital PCR plates (Applied Biosystems, Grand Island, NY, USA), and each sample was analyzed in duplicate.

### Data analysis

Data shown are means ± standard error of the mean. Statistical significance was estimated using one-way analysis of variance and post hoc tests as delineated in the legends as explained in the figure legends. The software (Graphpad 11) was used such that first the 2-way ANOVA interaction results were calculated, and when this led to significant group differences, the software, in the second step, calculated pairwise tests. Numbers in brackets in the figures indicate the number of patients investigated. A *p*-value smaller than 0.05 was considered to be significant.

### Drugs and materials

The drugs isoprenaline, forskolin, dibutyryl-cAMP, serotonin, semaglutide, histamine, acetylcholine, carbachol, pilocarpine, atropine, AF-DX 116 (AF-DX), fenoldopam and muscarine were purchased from Sigma-Aldrich (Taufkirchen, Germany) or Hycult (Beutelsbach, Germany). All other chemicals were of the highest purity grade commercially available. Deionized water was used throughout the experiments. Stock solutions were prepared fresh daily.

## Results

**Fig. 2:**
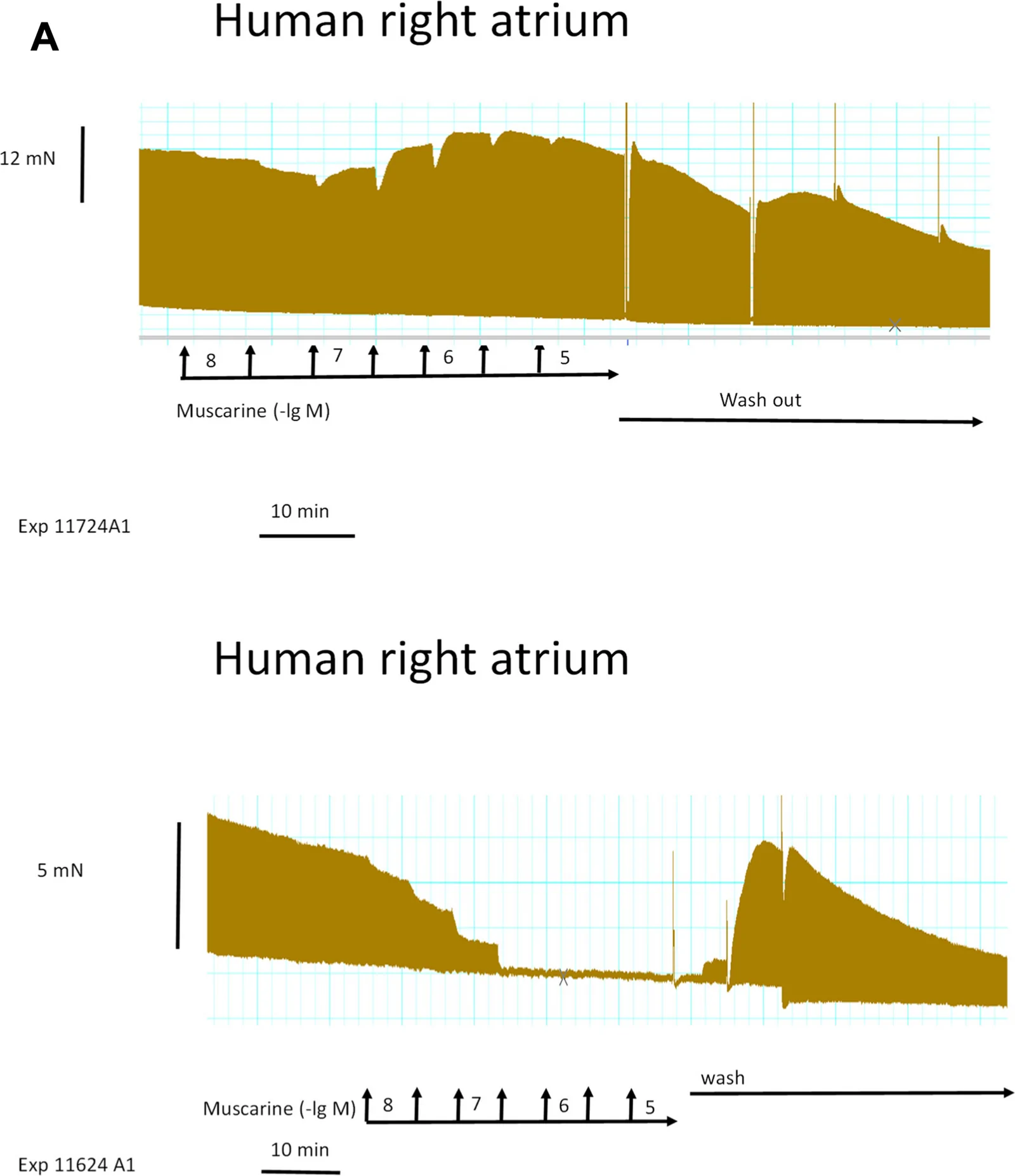

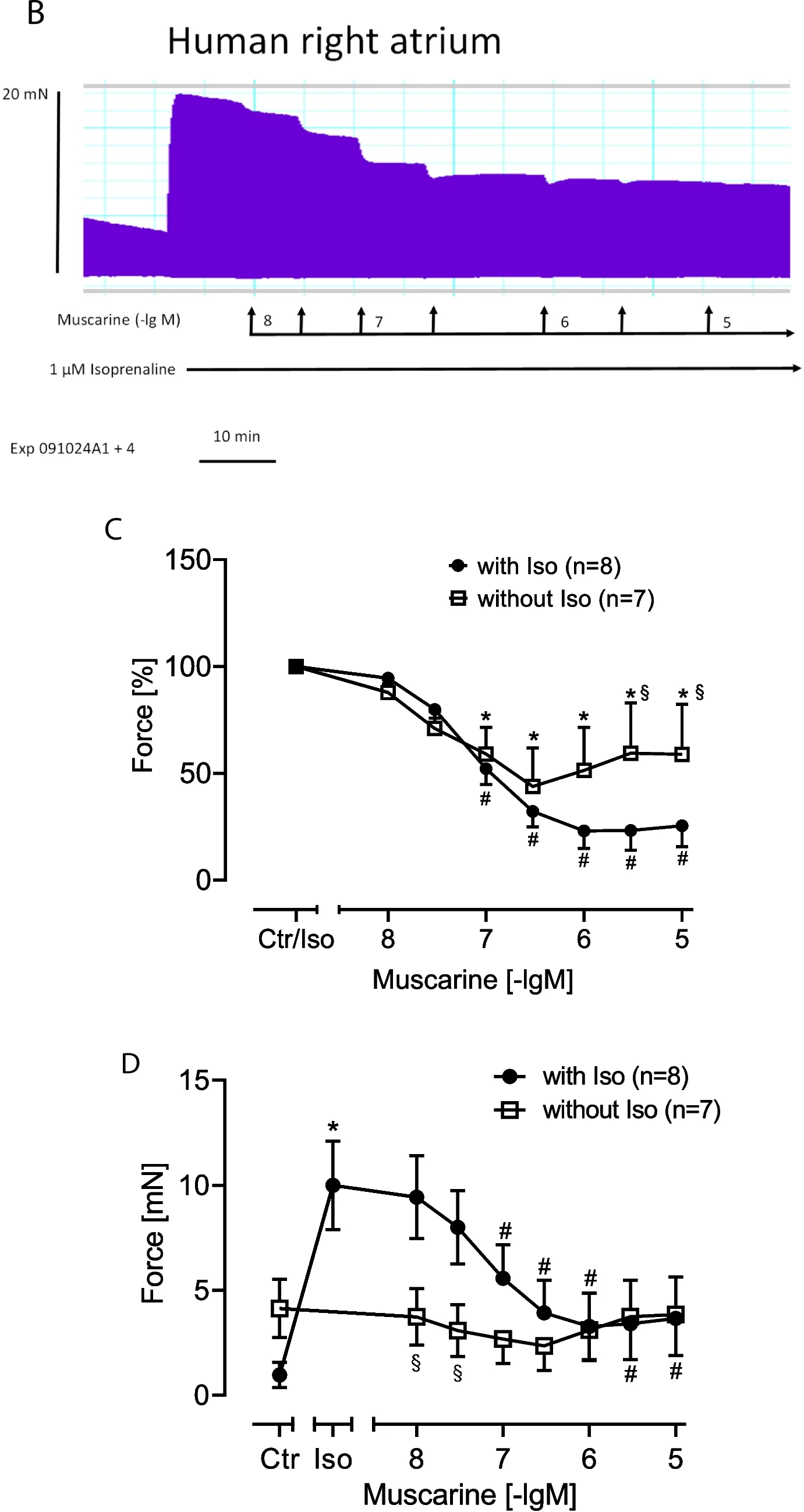
**A** Original recordings of inotropic effects of muscarine alone in two isolated electrically driven human right atrial preparations. Increasing concentrations of muscarine were cumulatively applied. Please note two patterns. In the first pattern (**A**, top), muscarine exerts an initial negative inotropic effect followed by a sustained slowly developing positive inotropic effect. In the second pattern (**A**, bottom), in a different patient (identified by experimental date below each recording), there was only a negative inotropic effect to muscarine. The ordinate gives the force of contraction in milliNewton (mN, ordinate) over time in minutes (horizontal bars, time in minutes (min)). The abscissa gives negative decadic logarithm molar concentrations (− lgM) of muscarine. **B** Original recordings of inotropic effects of muscarine in the presence of isoprenaline in isolated electrically driven human right-atrial preparations. First, isoprenaline increased the force of contraction. Then increasing concentrations of muscarine were cumulatively applied. Please note that muscarine exerts a sustained negative inotropic effect. The ordinate gives the force of contraction in mN (ordinate) over time in minutes (horizontal bar, time in minutes (min)). The abscissa gives negative decadic logarithm molar concentrations (− lgM) of muscarine. **C** Percentile effect of muscarine on the force of contraction in human atrial preparations. Ordinate indicates the force of contraction in percentage of pre-drug values (100% Ctr, 1 µM isoprenaline (Iso), respectively). The abscissa indicates Ctr (pre-drug value) or 1 µM isoprenaline and increasing concentrations of muscarine in negative decadic logarithmic molar concentrations (− lgM). Data are plotted as mean values and SEM. *p < 0.05 vs. Ctr; ^#^*p* < 0.05 vs. Iso (2-way ANOVA with Bonferroni’s multiple comparison test); ^§^*p* < 0.05 vs. with Iso (pairwise comparison with Fisher’s least significant difference (LSD) test. More specifically, the force values after agonist application obtained in the absence of isoprenaline (without Iso) were compared to their own pre-drug values (before application of agonist) presence. When isoprenaline was first given (with Iso), the force induced by agonist concentrations was tested against the pre-force value that had been increased by isoprenaline. Numbers in brackets indicate the number of patients studied. **D** Absolute effect of muscarine on the force of contraction in human atrial preparations. Ordinate indicates the force of contraction in mN. The abscissa indicates Ctr (pre-drug value) or 1 µM isoprenaline (Iso) and concentrations of muscarine in negative decadic logarithm molar concentrations (− lgM). Data are plotted as mean values and SEM. *p < 0.05 vs. Ctr; ^#^*p* < 0.05 vs. Iso (2-way ANOVA with Bonferroni’s multiple comparison test); ^§^*p* < 0.05 vs. with Iso (pairwise comparison with Fisher’s least significant difference (LSD) test). More specifically, the force values after agonist application obtained in the absence of isoprenaline (without Iso) were compared to their own pre-drug values (before application of agonist) presence. When isoprenaline was first given (with Iso), the force induced by agonist concentrations was tested against the pre-force value that had been increased by isoprenaline. Numbers in brackets indicate the number of patients studied

We first studied the direct and indirect effects of muscarine. Original recordings show the effects of cumulatively applied muscarine alone (Fig. 2A). As exemplified in Fig. 2A (top), we noted in some patients a concentration-dependent negative inotropic effect followed by a positive inotropic effect. As exemplified in Fig. 2A (bottom), we noted in other patients only a concentration-dependent negative inotropic effect. The initial negative inotropic effect is summarized in Fig. 2C in percentage and in Fig. 2D in absolute values of developed force (open squares). In some samples (Fig. 2A, top), there was a remarkable positive inotropic effect to muscarine which was completely lacking in other samples (Fig. 2A, bottom). This probably explains the wide scatter of the data in Fig. 2C. Apparently, two patterns of responses to muscarine exist and they are patient dependent.

Isoprenaline increases the force of contraction (Fig. 2D). This positive inotropic effect of isoprenaline is not visible in Fig. 2C, because we normalized values before muscarine in the absence (Ctr) and presence of isoprenaline (Iso) as 100%. In Fig. 2C the negative inotropic effect of low concentrations of muscarine (below 100 nM) is similar in the presence and absence of isoprenaline. After isoprenaline (Fig. 2B), we mainly noted a negative inotropic effect of muscarine as summarized in Fig. 2C. Please note that in Fig. 2A, at high concentrations of muscarine, there was a positive inotropic effect. When we plot the maximum negative inotropic effects after muscarine addition in the presence of isoprenaline, a smooth curve emerges (Fig. 2C and D, closed circles). The plot of the absolute values depicts how effective muscarine acts at elevated contractile values (i.e., in the presence of isoprenaline, Fig. 2D, closed circles).

**Fig. 3:**
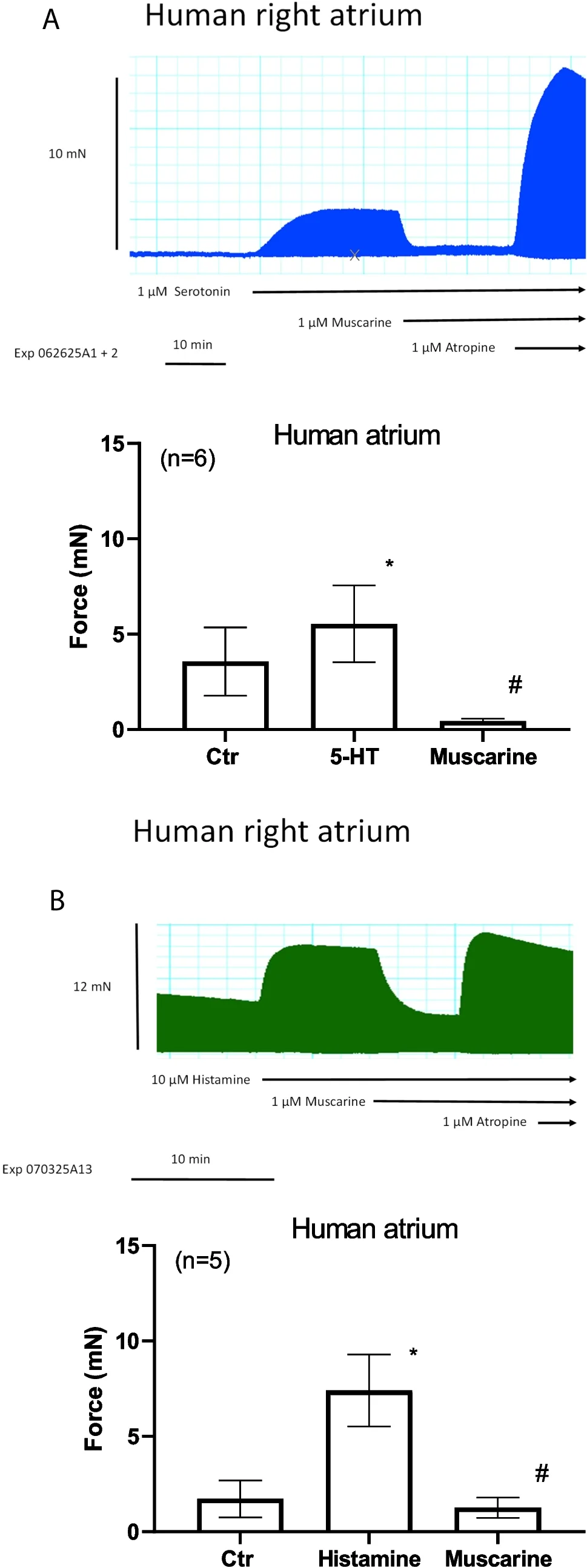

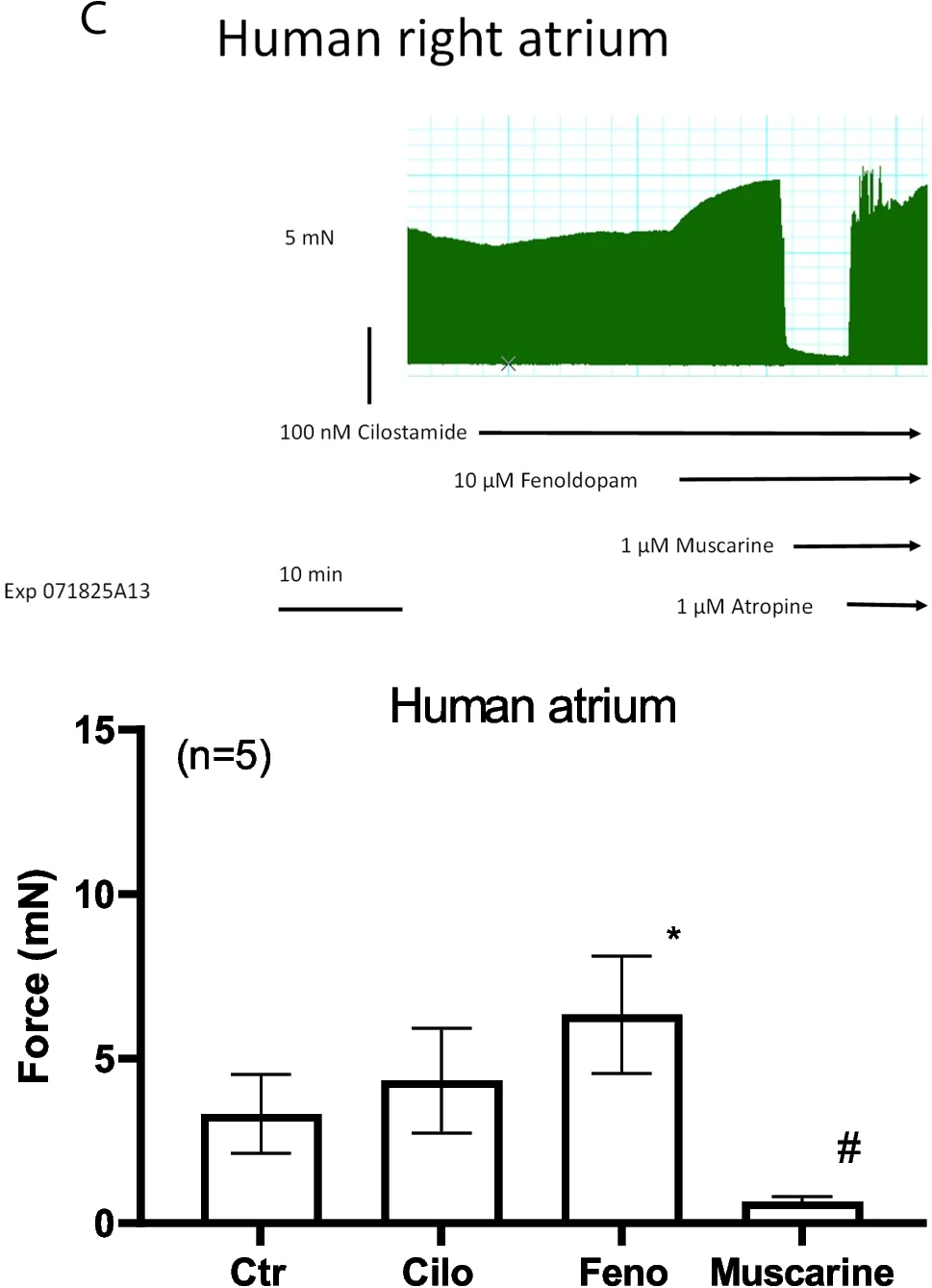
**A** Muscarine reduces the positive inotropic effects of serotonin**.** Original recordings (top) of inotropic effects of muscarine in the presence of serotonin in isolated electrically driven human right atrial preparations. First, serotonin (5-HT) increased the force of contraction. Then 1 µM muscarine was applied. Muscarine rapidly reduces force, and this effect is reversed by atropine. Summarized bar diagram (bottom) for the force of contraction under pre-drug conditions (Ctr), the maximum positive inotropic effect of 1 µM serotonin, and the effect of additionally applied 1 µM muscarine. These conditions were compared using ANOVA, and Bonferroni’s test was used to compare between relevant conditions. Numbers in brackets indicate the number of experiments. *P* < 0.05 is indicated with an asterisk (*) or a number sign (#) against Ctr or 5-HT, respectively. The ordinates give the force of contraction in mN. The horizontal bar indicates time in minutes (min). Numbers in brackets indicate the number of patients studied. **B** Muscarine reduces the positive inotropic effects of histamine. Original recordings (top) of inotropic effects of muscarine in the presence of histamine in isolated electrically driven human right atrial preparations. First, histamine increased the force of contraction. Then 1 µM muscarine was applied. Muscarine rapidly reduced force. Summarized bar diagram (bottom) for the force of contraction under pre-drug conditions (Ctr), the maximum positive inotropic effect of 10 µM histamine, and the effect of additionally applied 1 µM muscarine. These conditions were compared using ANOVA, and Bonferroni’s test was used to compare between relevant conditions. Numbers in brackets indicate the number of experiments. *P* < 0.05 is indicated with an asterisk (*) or a number sign (#) against Ctr or histamine, respectively. The ordinates give the force of contraction in mN. The horizontal bar indicates time in minutes (min). Numbers in brackets indicate the number of patients studied. **C** Muscarine reduces the positive inotropic effects of fenoldopam. Original recordings (top) of inotropic effects of muscarine in the presence of fenoldopam in isolated electrically driven human right atrial preparations. First, fenoldopam (Feno) increased the force of contraction in the presence of cilostamide. Then 1 µM muscarine was applied. Muscarine rapidly reduced force. Summarized bar diagram (bottom) for the force of contraction under pre-drug conditions (Ctr), the maximum positive inotropic effect of 100 nM cilostamide, the maximum positive inotropic effect of additional fenoldopam (10 µM), and the effect of additionally applied 1 µM muscarine. These conditions were compared using ANOVA, and Bonferroni’s test was used to compare between relevant conditions. Numbers in brackets indicate the number of experiments. *P* < 0.05 is indicated an asterisk (*) or a number sign (#) against cilostamide or fenoldopam, respectively. The ordinates give the force of contraction in mN. The horizontal bar indicates time in minutes (min). Numbers in brackets indicate the number of patients studied

Next, the question arose how muscarine would act, if the force of contraction were not elevated by β-adrenoceptor stimulation (isoprenaline), but by activation of other monoamine sensitive receptors of the A class of G-protein-coupled receptors (Katritch et al. 2013). For example, the monoamine serotonin also increased force in the human atrium (via 5-HT_4_-receptors, Fig. 1, cf. Kaumann et al. 1990) and this effect was attenuated by muscarine (Fig. 3A top). The negative inotropic effect of muscarine after serotonin was reversed by atropine (Fig. 3A, top). This also indicates that muscarine has not damaged the muscle unspecifically but really acted via an atropine-sensitive receptor in a reversible way. The data from several experiments are summarized in Fig. 3A (bottom). Furthermore, histamine (10 µM) exerted a positive inotropic effect (Fig. 3B, top). Histamine acts via H_2_-histamine receptors to increase the force of contraction in the human atrium (Genovese et al. 1988). This positive inotropic effect of histamine was reversed by additionally applied muscarine (Fig. 3B top). The negative inotropic effect of histamine was reversed by atropine (Fig. 3B, top). The data on the negative inotropic effect of muscarine in the presence of histamine are summarized in Fig. 3B (bottom). Next, we had stimulated D_1_-dopamine receptors by fenoldopam. Fenoldopam augmented the force of contraction (Fig. 3C, top). Here, we pre-incubated the HAP with a low concentration of cilostamide because this is necessary to detect any positive inotropic effect after fenoldopam that stimulates D_1_-receptors in HAP (Gergs et al. 2024). This effect of muscarine at D_1_-receptors was likewise reversed by atropine (Fig. 3C, top). Data from such experiments with fenoldopam are summarized in Fig. 3C (bottom).

**Fig. 4:**
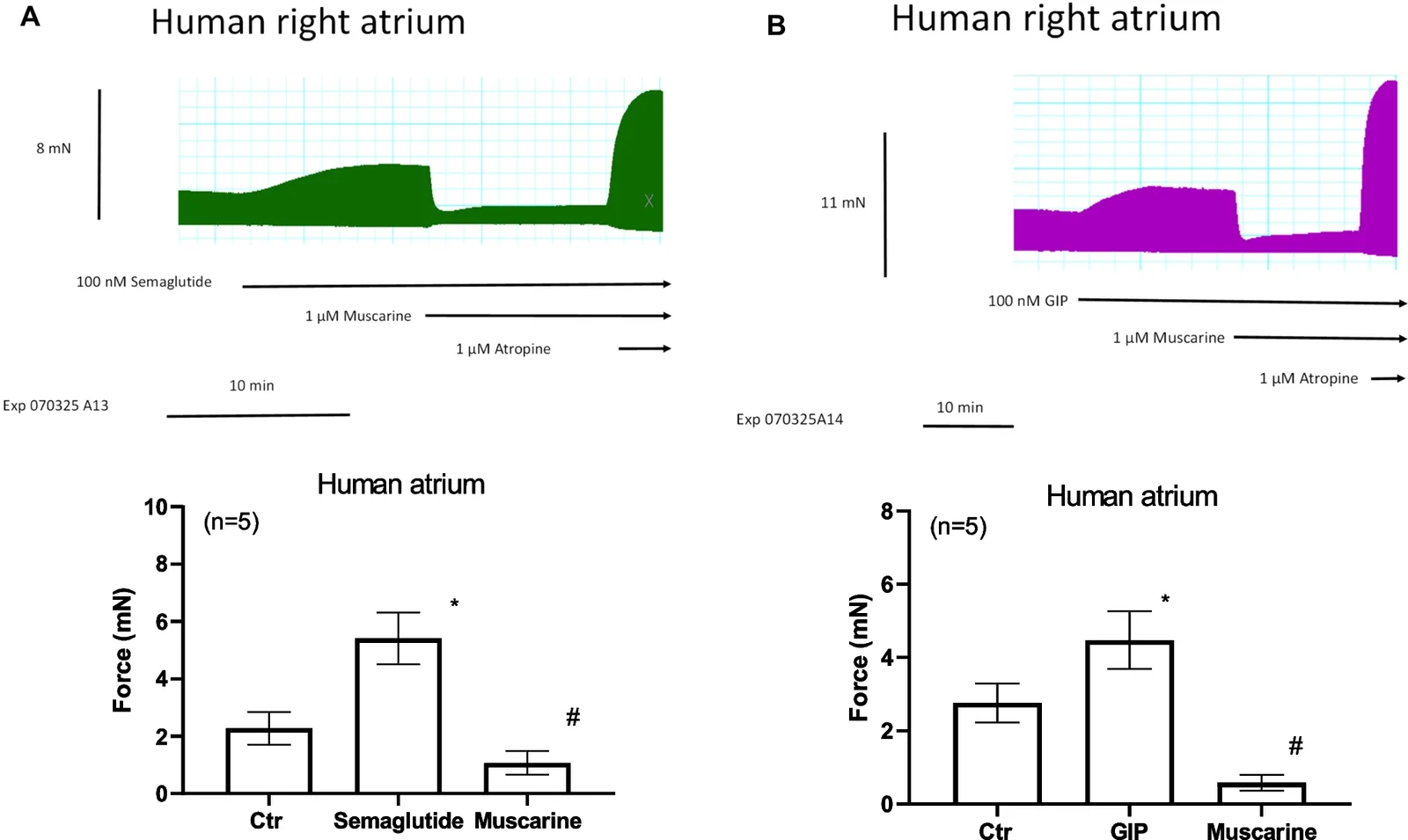
**A** Muscarine reduces the positive inotropic effects of semaglutide. Original recordings (top) of inotropic effects of muscarine in the presence of semaglutide in isolated electrically driven human right atrial preparations. First, semaglutide increased the force of contraction. Then 1 µM muscarine was applied. Muscarine rapidly reduced force. Summarized bar diagram (bottom) for the force of contraction under pre-drug conditions (Ctr), the maximum positive inotropic effect of 100 nM semaglutide, and the effect of additionally applied 1 µM muscarine. These conditions were compared using ANOVA, and Bonferroni’s correction was used for multiple comparisons. Numbers in brackets indicate the number of experiments. *P* < 0.05 is indicated an asterisk (*) or a number sign (#) against Ctr or semaglutide, respectively. The ordinates give the force of contraction in mN. The horizontal bar indicates time in minutes (min). Numbers in brackets indicate the number of patients studied. **B** Muscarine reduces the positive inotropic effects of GIP. Original recordings (top) of inotropic effects of muscarine in the presence of gastrin inhibitory peptide (GIP) in isolated electrically driven human right atrial preparations. First, GIP increased the force of contraction. Then, 1 µM muscarine was applied. Muscarine rapidly reduced force. Summarized bar diagram (bottom) for the force of contraction under pre-drug conditions (Ctr), the maximum positive inotropic effect of 100 nM GIP, and the effect of additionally applied 1 µM muscarine. These conditions were compared using ANOVA, and Bonferroni’s correction was used for multiple comparisons. Numbers in brackets indicate the number of experiments. *P* < 0.05 is indicated an asterisk (*) or a number sign (#) against Ctr or GIP, respectively. The ordinates give the force of contraction in mN. The horizontal bar indicates time in minutes (min). Numbers in brackets indicate the number of patients studied

To study other receptors from the class B of G-protein-coupled receptors (Katritch et al. 2013) that are known to increase cAMP in the heart, we also studied semaglutide, an agonist at glucagon-like-peptide-1 receptors (Fig. 1). Semaglutide raised the force of contraction, as was shown before (Neumann et al. 2024). That is plotted in an original registration in Fig. 4A (top). Additionally applied muscarine reduced the force of contraction (Fig. 4A, top). These experiments were summarized in Fig. 4B (bottom). In addition, we decided to stimulate the force of contraction with gastric inhibitory peptide (glucose-dependent insulinotropic peptide, GIP), which acts via the GIP receptor (class B of G-protein-coupled receptors). We have reported before that GIP can increase the force of contraction in the isolated human atrium (Neumann et al. 2025). Indeed, GIP increased the force of contraction and this effect was also reversed by muscarine. This is depicted in an original recording (Fig. 4, top) and also in a bar diagram (Fig. 4B, bottom). Hence, muscarine reversed the positive inotropic effects of drugs that are known to elevate cAMP via class A and class B G-protein-coupled receptors.

**Fig. 5:**
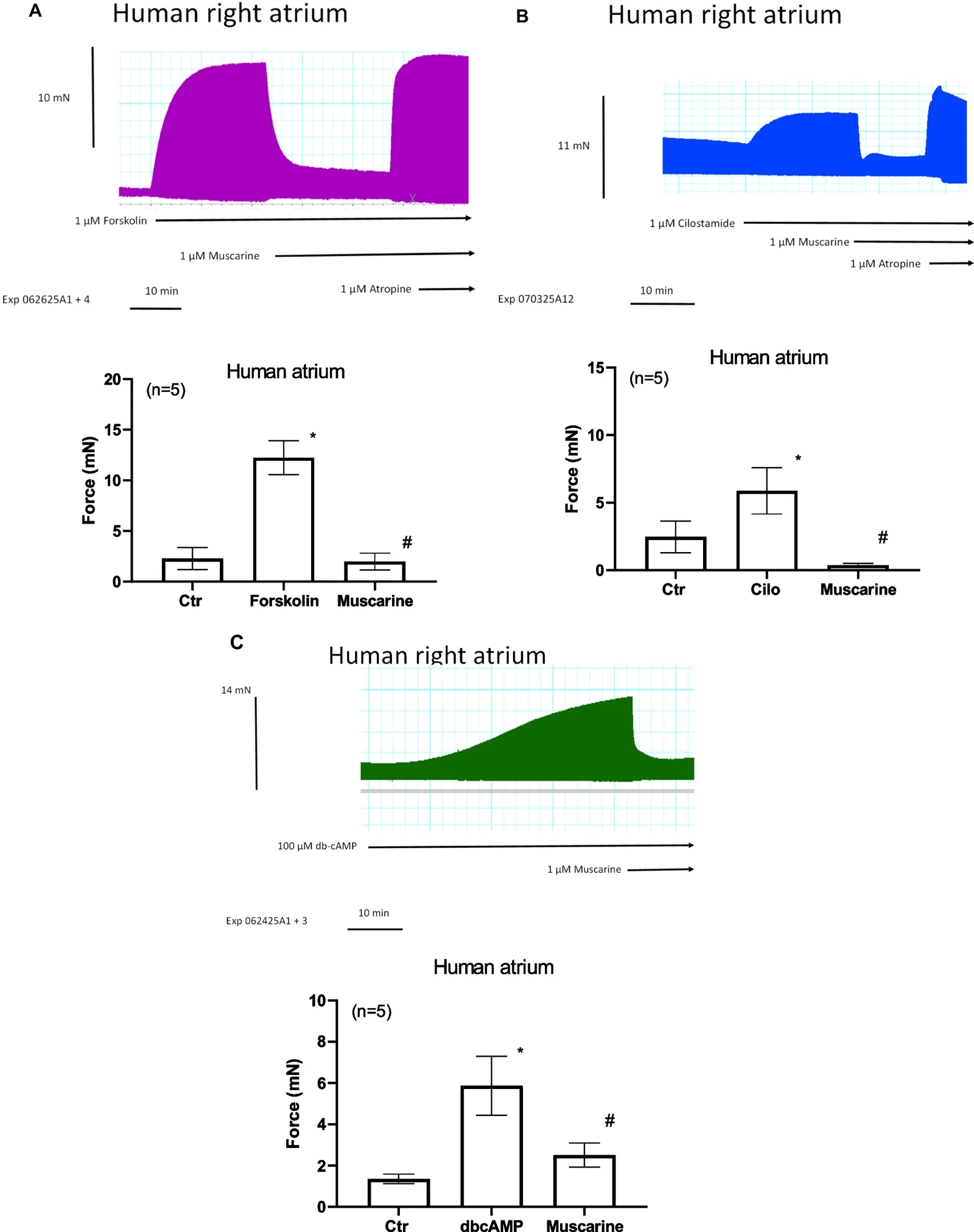
**A** Muscarine reduces the positive inotropic effects of forskolin. Original recordings (top) of inotropic effects of muscarine in the presence of forskolin in isolated electrically driven human right atrial preparations. First, forskolin increased the force of contraction. Then 1 µM muscarine was applied. Muscarine rapidly reduced force. Summarized bar diagram (bottom) for the force of contraction under pre-drug conditions (Ctr), the maximum positive inotropic effect of 1 µM forskolin, and the effect of additionally applied 1 µM muscarine. These conditions were compared using ANOVA, and Bonferroni’s correction was used for multiple comparisons. Numbers in brackets indicate the number of experiments. *P* < 0.05 is indicated an asterisk (*) or a number sign (#) against Ctr or forskolin, respectively. The ordinates give the force of contraction in mN. The horizontal bar indicates time in minutes (min). Numbers in brackets indicate the number of patients studied. **B** Muscarine reduces the positive inotropic effects of cilostamide. Original recordings (top) of inotropic effects of muscarine in the presence of cilostamide in isolated electrically driven human right atrial preparations. First, 1 µM cilostamide (Cilo) increased the force of contraction. Then 1 µM muscarine was applied. Muscarine rapidly reduced force. Summarized bar diagram (bottom) for the force of contraction under pre-drug conditions (Ctr), the maximum positive inotropic effect of 1 µM cilostamide, and the effect of additionally applied 1 µM muscarine. These conditions were compared using ANOVA, and Bonferroni’s correction was used for multiple comparisons. Numbers in brackets indicate the number of experiments. *P* < 0.05 is indicated an asterisk (*) or a number sign (#) against Ctr or cilostamide, respectively. The ordinates give the force of contraction in mN. The horizontal bar indicates time in minutes (min). Numbers in brackets indicate the number of patients studied. **C** Muscarine reduces the positive inotropic effects of dibutyryl-cAMP. Original recordings (top) of inotropic effects of muscarine in the presence of dibutyryl-cAMP in isolated electrically driven human right atrial preparations. First, dibutyryl-cAMP (db-cAMP) increased the force of contraction. Then 1 µM muscarine was applied. Muscarine rapidly reduced force. Summarized bar diagram (bottom) for the force of contraction under pre-drug conditions (Ctr), the maximum positive inotropic effect of 100 µM dbcAMP, and the effect of additionally applied 1 µM muscarine. These conditions were compared using ANOVA, and Bonferroni’s correction was used for multiple comparisons. Numbers in brackets indicate the number of experiments. *P* < 0.05 is indicated an asterisk (*) or a number sign (#) against Ctr or dbcAMP, respectively. The ordinates give the force of contraction in mN. The horizontal bar indicates time in minutes (min). Numbers in brackets indicate the number of patients studied

To better understand the underlying mechanism of the negative inotropic effect of muscarine in the presence of elevated cAMP or an enhanced activity of the cAMP-dependent protein kinase, we systematically used further tools. First, we directly stimulated the activity of adenylyl cyclase in HAP with forskolin. Forskolin increased the force of contraction (Fig. 5A, top). Of note, muscarine reversed the positive inotropic effects of forskolin (Fig. 5A, top). Forskolin is a naturally occurring compound that stimulates the adenylyl cyclase independently of any receptor occupation (Fig. 1, e.g., Schwarz et al. 2025). This negative inotropic effect of muscarine was also reversed by atropine (Fig. 5A, top). Such data are summarized in (Fig. 5A, bottom). Hence, muscarine is unlikely to directly inhibit the adenylyl cyclase activity. Another way to increase cAMP levels, independent of any receptor activation, is the inhibition of the degradation of cAMP by phosphodiesterases (Fig. 1, e.g., Rayo-Abella et al. 2023; Schwarz et al. 2025). Therefore, we studied 1 µM cilostamide, a concentration which clearly raised the force of contraction in HAP (Fig. 5B, top). Interestingly, muscarine also exerted a negative inotropic effect after cilostamide, a phosphodiesterase III inhibitor (Fig. 5B). Hence, muscarine seems not to reduce the force of contraction by, e.g., stimulation of the activity of phosphodiesterase III. One can see this in an original recording (Fig. 5B, top) and this effect is summarized in Fig. 5B (bottom). Furthermore, we directly (independent of a receptor) activated the cAMP-dependent protein kinase with the cell-membrane permeant drug dibutyryl-cAMP (Fig. 1). The dibutyryl-cAMP raised the force of contraction slowly (Fig. 5C, top) as in a previous report (Schwarz et al. 2024). Additionally applied muscarine exerted a negative inotropic effect in the presence of dibutyryl-cAMP. This is depicted in an original recording (Fig. 5C, top) and summarized below (Fig. 5C, bottom). These data might indicate that muscarine acts via additional mechanisms such as an activation of phosphatase(s), as we had suggested for carbachol (Schwarz et al. 2023).

**Fig. 6:**
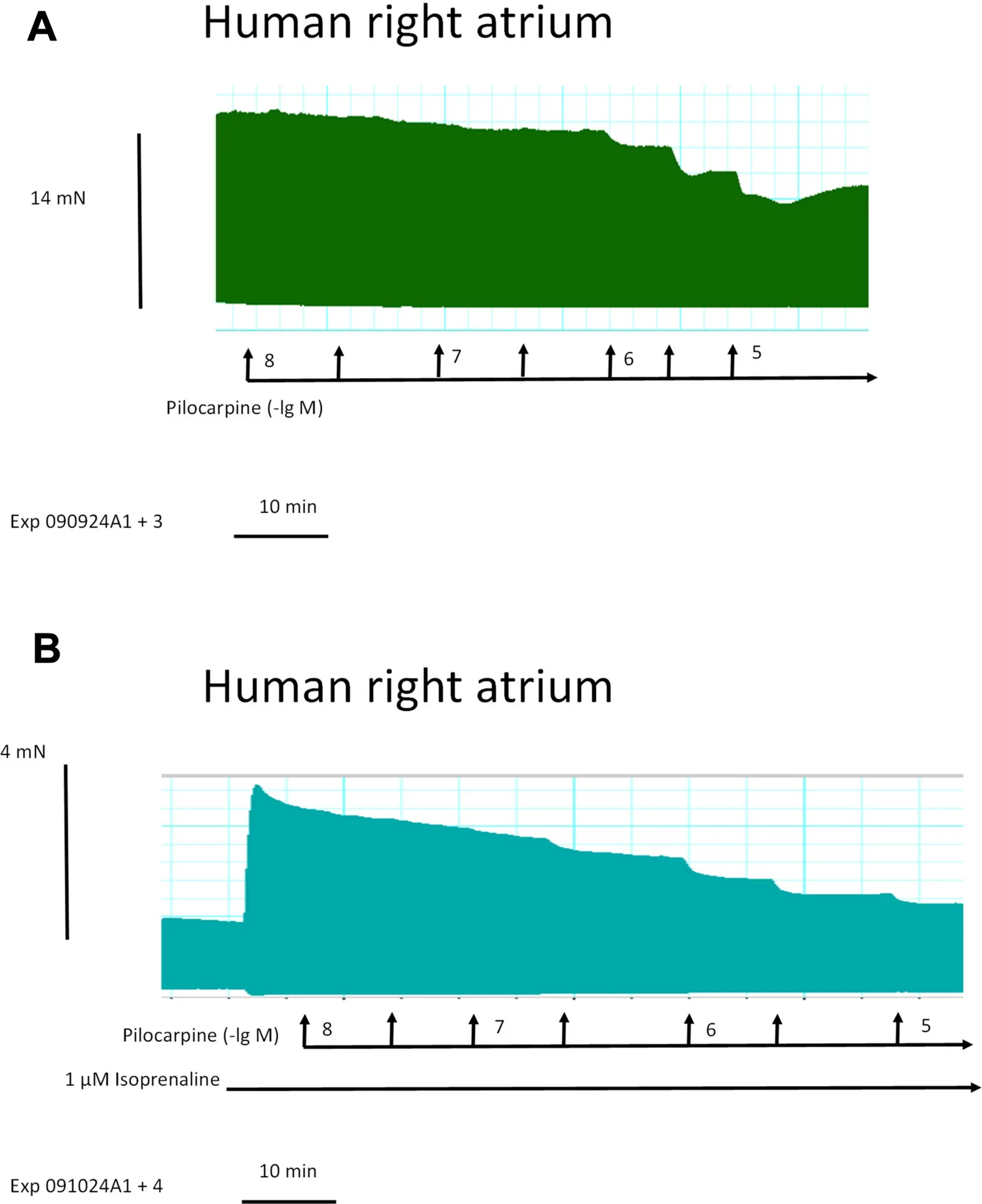

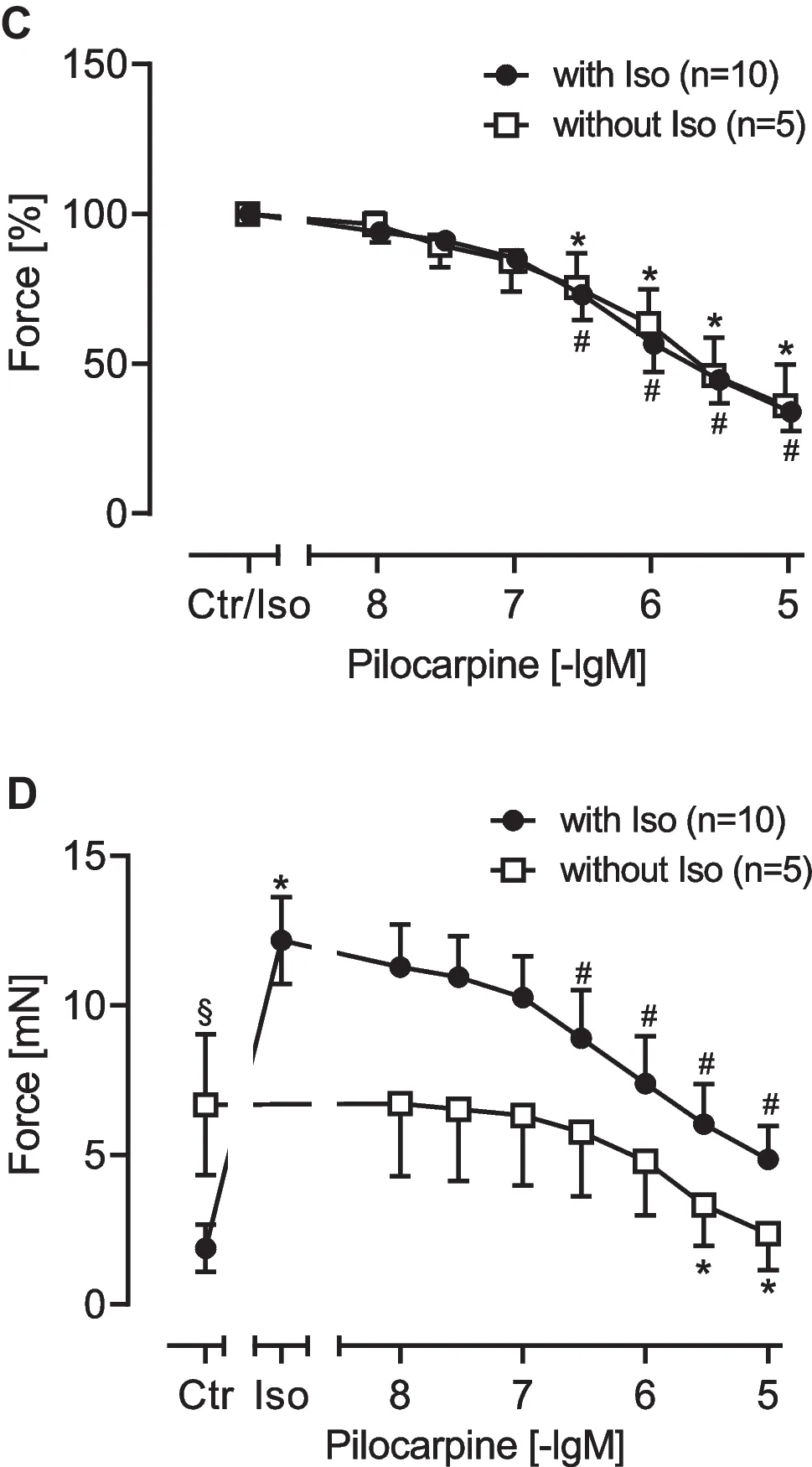
**A** Original recordings of inotropic effects of pilocarpine alone in isolated electrically driven human right atrial preparations. Increasing concentrations of muscarine (in lg molar concentrations) were cumulatively applied. Please note that muscarine exerts an initial negative inotropic effect followed by a sustained slowly developing positive inotropic effect. The ordinate gives the force of contraction in mN (ordinate) over time in minutes (horizontal bars, time in minutes (min)). The abscissa gives the negative decadic logarithm molar concentrations (− lgM) of pilocarpine. **B** Original recordings of inotropic effects of pilocarpine in the presence of isoprenaline in isolated electrically driven human right atrial preparations. The first isoprenaline increased force. Increasing concentrations of muscarine (in lg molar concentrations) were cumulatively applied. Please note that muscarine exerts a sustained slowly developing positive inotropic effect. The ordinate gives the force of contraction in mN (ordinate) over time in minutes (horizontal bars, time in minutes (min)). The abscissa gives negative decadic logarithm molar concentrations (− lgM) of pilocarpine. **C** Percentile effect of pilocarpine on the force of contraction in human atrial preparations. The ordinate indicates the force of contraction in percentage of pre-drug values (100% Ctr, 1 µM isoprenaline (Iso), respectively). The abscissa indicates Ctr (pre-drug value) or 1 µM isoprenaline and increasing concentrations of pilocarpine in negative decadic logarithmic molar concentrations (− lgM). Data are plotted as mean values and SEM. *p < 0.05 vs. Ctr; ^#^*p* < 0.05 vs. Iso (2-way ANOVA with Bonferroni’s multiple comparison test). More specifically, the force values after agonist application obtained in the absence of isoprenaline (without Iso) were compared to their own pre-drug values (before application of agonist) presence. When isoprenaline was first given (with Iso), the force induced by agonist concentrations was tested against the pre-force value that had been increased by isoprenaline. Numbers in brackets indicate the number of patients studied. **D** Absolute effect of pilocarpine on the force of contraction in human atrial preparations. Ordinate indicates the force of contraction in mN. The abscissa indicates Ctr (pre-drug value) or 1 µM isoprenaline (Iso) and concentrations of pilocarpine in negative decadic logarithm molar concentrations (− lgM). Data are plotted as mean values and SEM. *p < 0.05 vs. Ctr; ^#^*p* < 0.05 vs. Iso (2-way ANOVA with Bonferroni’s multiple comparison test); ^§^*p* < 0.05 vs. with Iso (pairwise comparison with Fisher’s least significant difference (LSD) test). More specifically, the force values after agonist application obtained in the absence of isoprenaline (without Iso) were compared to their own pre-drug values (before application of agonist) presence. When isoprenaline was first given (with Iso), the force induced by agonist concentrations was tested against the pre-force value that had been increased by isoprenaline. Numbers in brackets indicate the number of patients studied

Next, we wanted to compare the effects of muscarine in HAP with those of other well-known muscarinic receptor agonists. We first studied pilocarpine. Pilocarpine has a negative inotropic effect (Fig. 6A, C, and D). This effect of pilocarpine is further shifted to the left to demonstrate heightened sensitivity in the presence of isoprenaline (Fig. 6D). Several experiments on pilocarpine alone are summarized in Fig. 6C in percentages, and in Fig. 6D, in absolute values of developed force (open squares). In Fig. 6C, using an ordinate in percentages, the negative inotropic effects of pilocarpine in the absence and presence of isoprenaline are superimposable. However, this presentation (Fig. 6C) lost some information. When one regards Fig. 6D (depicting absolute force measurements), the effect of pilocarpine gains significance at lower concentrations in the presence of isoprenaline (closed circles) than in the absence of isoprenaline (open squares).

**Fig. 7:**
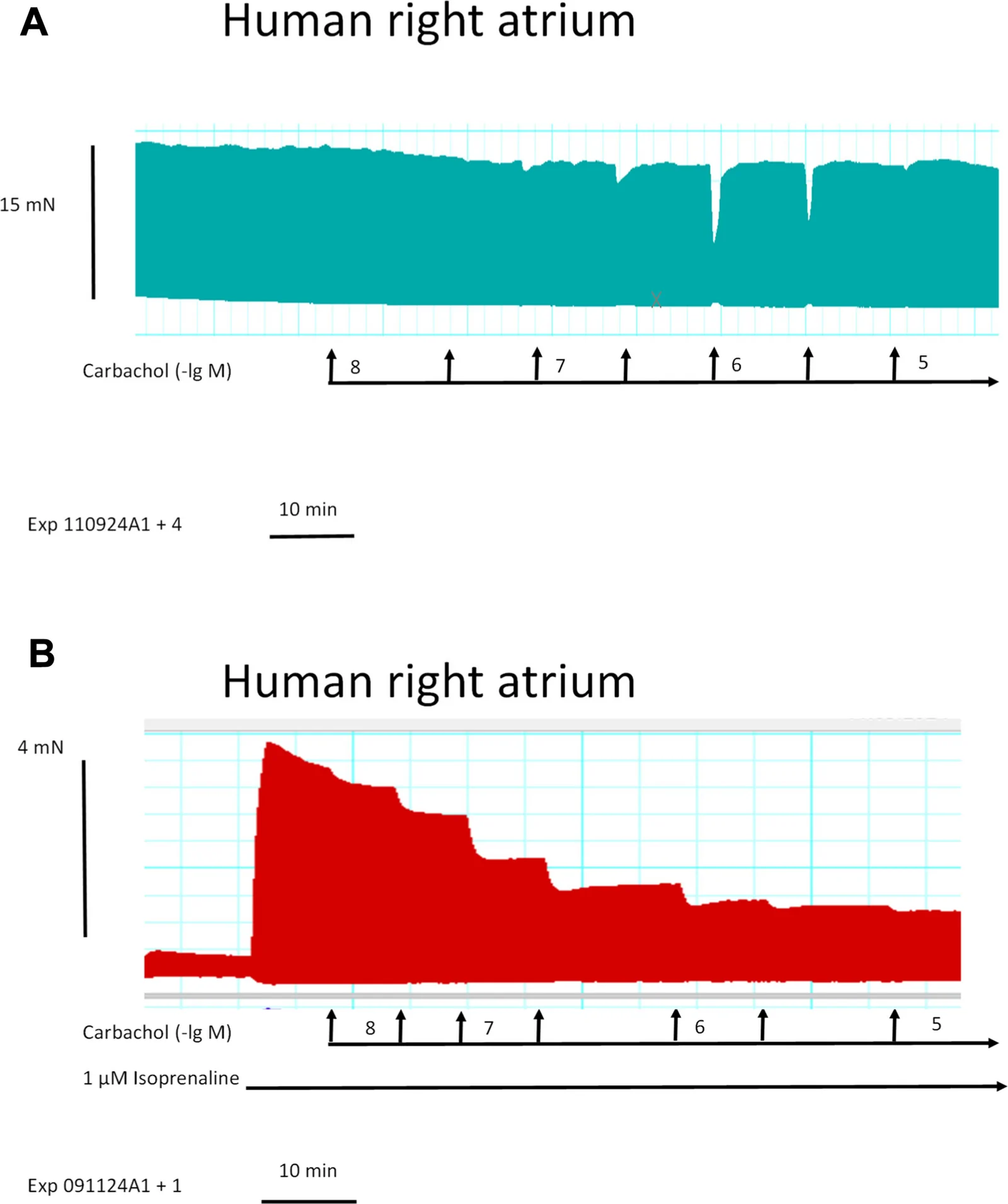

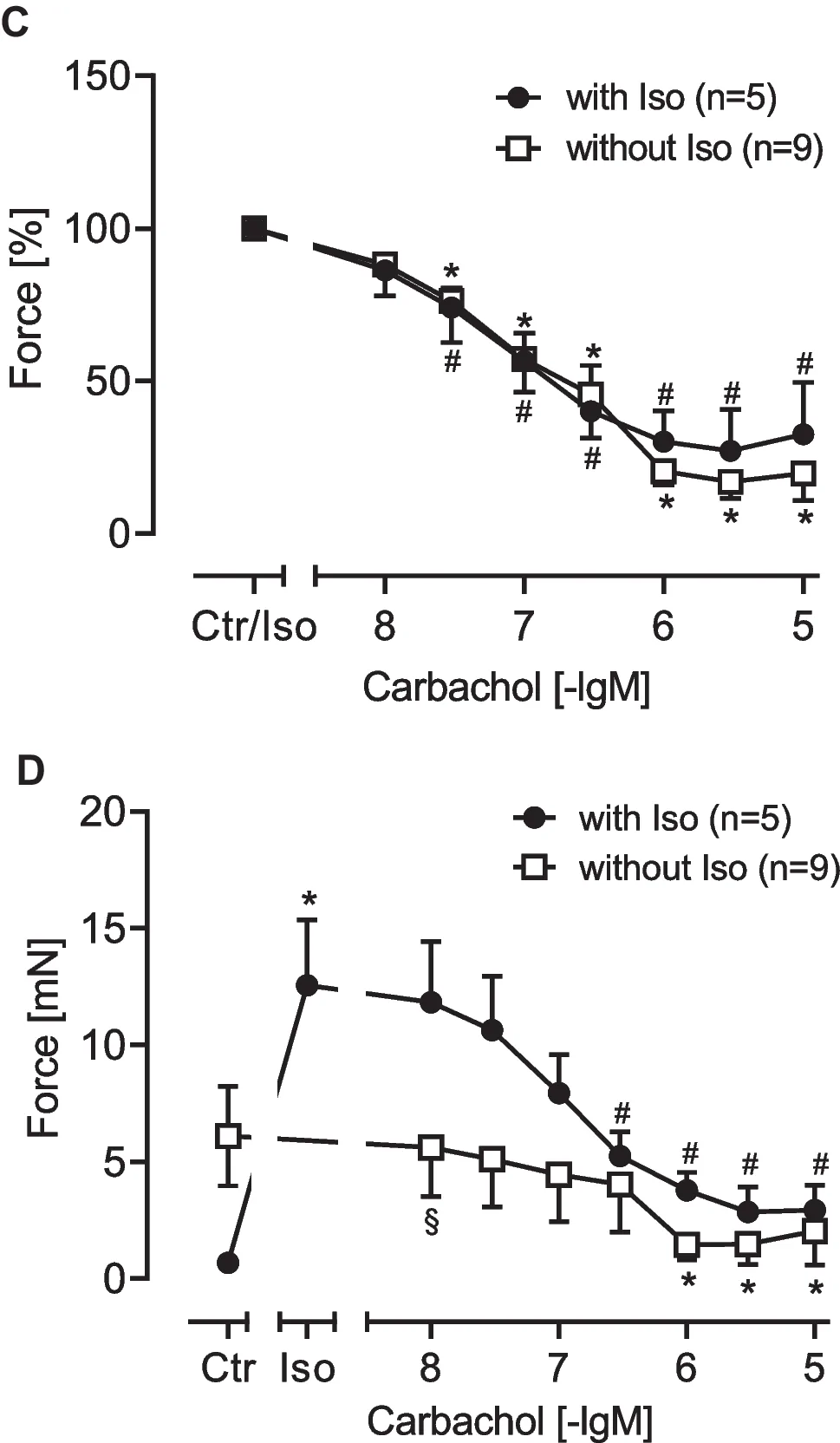
**A** Original recordings of inotropic effects of carbachol in isolated electrically driven human right atrial preparations. First isoprenaline application increased the force. Increasing concentrations of carbachol (in lg molar concentrations) were cumulatively applied. Please note that carbachol exerts a sustained slowly developing positive inotropic effect. The ordinate gives the force of contraction in mN (ordinate) over time in minutes (horizontal bars, time in minutes (min)). The abscissa gives negative decadic logarithm molar concentrations (− lgM) of carbachol. **B** Original recordings of inotropic effects of carbachol in the presence of isoprenaline in isolated electrically driven human right atrial preparations. First isoprenaline increased force. Increasing concentrations of carbachol (in lg molar concentrations) were cumulatively applied. Please note that carbachol exerts a sustained slowly developing positive inotropic effect. The ordinate gives the force of contraction in mN (ordinate) over time in minutes (horizontal bars, time in minutes (min)). The abscissa gives negative decadic logarithm molar concentrations (− lgM) of carbachol. **C** Percentile effect of carbachol on the force of contraction in human atrial preparations. Ordinate indicates the force of contraction in percentage of pre-drug values (100% Ctr, 1 µM isoprenaline (Iso), respectively). The abscissa indicates Ctr (pre-drug value) or 1 µM isoprenaline and increasing concentrations of carbachol in negative decadic logarithmic molar concentrations (− lgM). Data are plotted as mean values and SEM. *p < 0.05 vs. Ctr; ^#^*p* < 0.05 vs. Iso (2-way ANOVA with Bonferroni’s multiple comparison test). More specifically, the force values after agonist application obtained in the absence of isoprenaline (without Iso) were compared to their own pre-drug values (before application of agonist) presence. When isoprenaline was first given (with Iso), the force induced by agonist concentrations was tested against the pre-force value that had been increased by isoprenaline. Numbers in brackets indicate the number of patients studied. **D** Absolute effect of carbachol on the force of contraction in human atrial preparations. Ordinate indicates the force of contraction in mN. The abscissa indicates Ctr (pre-drug value) or 1 µM isoprenaline (Iso) and concentrations of carbachol in negative decadic logarithm molar concentrations (− lgM). Data are plotted as mean values and SEM. *p < 0.05 vs. Ctr; ^#^*p* < 0.05 vs. Iso (2-way ANOVA with Bonferroni’s multiple comparison test); ^§^*p* < 0.05 vs. with Iso (pairwise comparison with Fisher’s least significant difference (LSD) test). More specifically, the force values after agonist application obtained in the absence of isoprenaline (without Iso) were compared to their own pre-drug values (before application of agonist) presence. When isoprenaline was first given (with Iso), the force induced by agonist concentrations was tested against the pre-force value that had been increased by isoprenaline. Numbers in brackets indicate the number of patients studied

Pilocarpine is of clinical relevance in ophthalmology but is rarely used in cardiac pharmacology to study M-cholinoceptors. Therefore, we also tested two widely used M-cholinoceptor agonists (namely acetylcholine and carbachol) and wanted to compare their effects alone and after isoprenaline with those of muscarine. In fact, carbachol, as expected, alone reduced the force of contraction in HAP as seen in an original experiment (Fig. 7A). A negative inotropic effect was followed by a positive inotropic effect (Fig. 7A). The maximum negative inotropic effects of carbachol alone are summarized in Fig. 7C and D (open squares). If carbachol was given in the presence of isoprenaline (Fig. 7B for an original recording), then carbachol reduced the force of contraction in HAP. These effects are also summarized in Fig. 7C and D (closed circles). Figure 7D offers the possibility to assess the extent of the positive inotropic effect of isoprenaline. In the presence of isoprenaline, the negative inotropic effect of muscarine gains significance at lower concentrations (closed circles) than in the absence of isoprenaline (open squares, Fig. 7D).

**Fig. 8:**
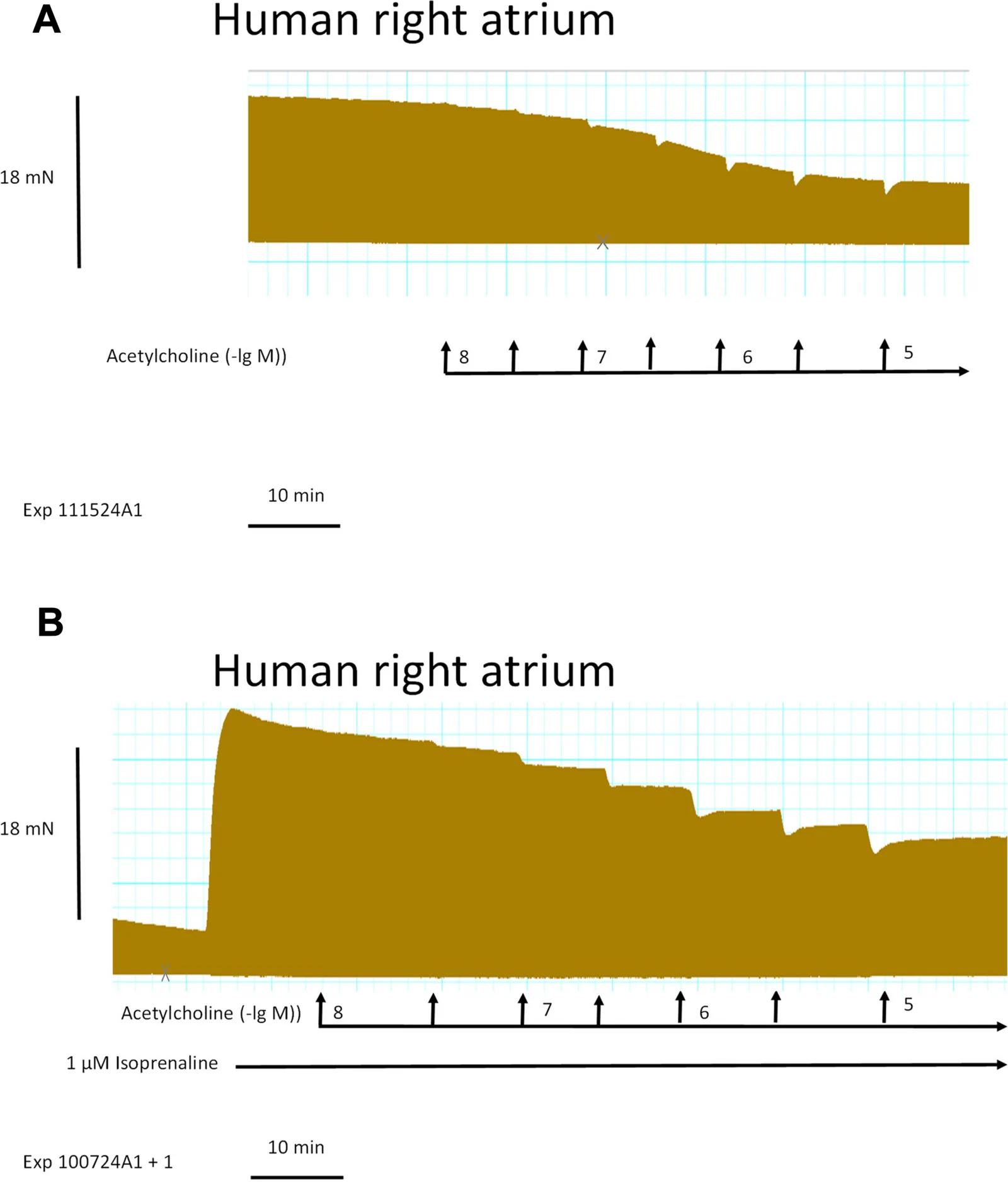

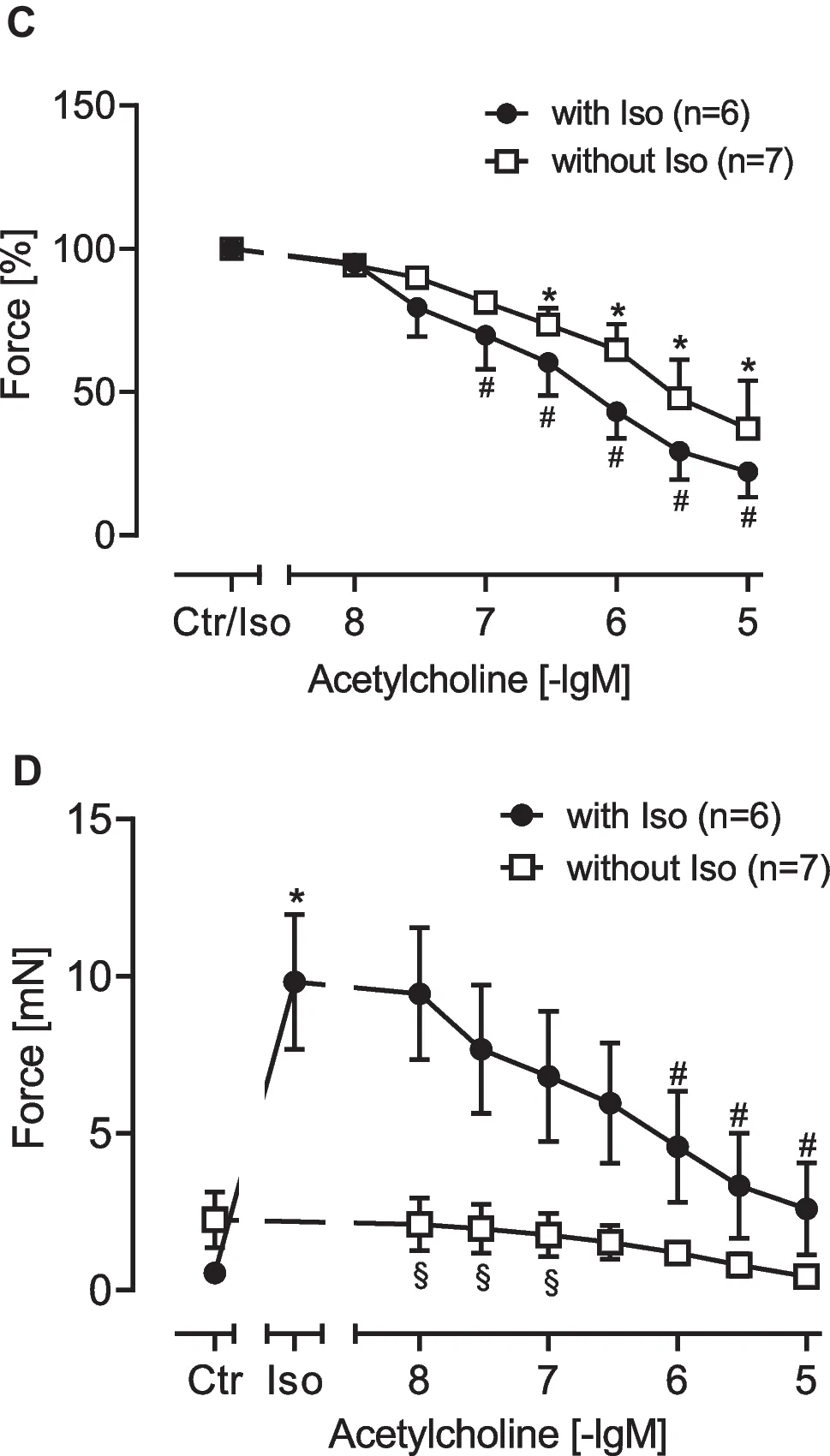
**A** Original recordings of inotropic effects of acetylcholine in isolated electrically driven human right atrial preparations. First isoprenaline increased force. Increasing concentrations of carbachol (in lg molar concentrations) were cumulatively applied. Please note that acetylcholine exerts a sustained slowly developing positive inotropic effect. The ordinate gives the force of contraction in mN (ordinate) over time in minutes (horizontal bars, time in minutes (min)). The abscissa gives concentrations of muscarine. **B** Original recordings of inotropic effects of acetylcholine in the presence of isoprenaline in isolated electrically driven human right atrial preparations. First isoprenaline increased force. Increasing concentrations of acetylcholine (in lg molar concentrations) were cumulatively applied. Please note that acetylcholine exerts a sustained slowly developing positive inotropic effect. The ordinate gives the force of contraction in mN (ordinate) over time in minutes (horizontal bars, time in minutes (min)). The abscissa gives concentrations of muscarine. **C** Percentile effect of acetylcholine on the force of contraction in human atrial preparations. Ordinate indicates the force of contraction in percentage of pre-drug values (100% Ctr, 1 µM isoprenaline (Iso), respectively). The abscissa indicates Ctr (pre-drug value) or 1 µM isoprenaline and increasing concentrations of acetylcholine in negative decadic logarithmic molar concentrations (− lgM). Data are plotted as mean values and SEM. *p < 0.05 vs. Ctr; ^#^*p* < 0.05 vs. Iso (2-way ANOVA with Bonferroni’s multiple comparison test). More specifically, the force values after agonist application obtained in the absence of isoprenaline (without Iso) were compared to their own pre-drug values (before application of agonist) presence. When isoprenaline was first given (with Iso), the force induced by agonist concentrations was tested against the pre-force value that had been increased by isoprenaline. Numbers in brackets indicate the number of patients studied. **D** Absolute effect of acetylcholine on the force of contraction in human atrial preparations. Ordinate indicates the force of contraction in mN. The abscissa indicates Ctr (pre-drug value) or 1 µM isoprenaline (Iso) and concentrations of acetylcholine in negative decadic logarithm molar concentrations (− lgM). Data are plotted as mean values and SEM. *p < 0.05 vs. Ctr; ^#^*p* < 0.05 vs. Iso (2-way ANOVA with Bonferroni’s multiple comparison test); ^§^*p* < 0.05 vs. with Iso (pairwise comparison with Fisher’s least significant difference (LSD) test). More specifically, the force values after agonist application obtained in the absence of isoprenaline (without Iso) were compared to their own pre-drug values (before application of agonist) presence. When isoprenaline was first given (with Iso), the force induced by agonist concentrations was tested against the pre-force value that had been increased by isoprenaline. Numbers in brackets indicate the number of patients studied

Finally, acetylcholine, the physiological agonist at all M-cholinoceptors, was studied. The pattern of the negative inotropic effect of acetylcholine on HAP in the absence and in the presence of isoprenaline was similar but not identical to that of carbachol. There was a negative inotropic effect, but at each concentration of acetylcholine, this was followed by only a small positive inotropic effect (Fig. 8A and B). Again, we plotted the maximum negative inotropic effects of acetylcholine alone and in the presence of isoprenaline. This was done in percentage terms (Fig. 8C) but also in absolute values for the force of contraction (Fig. 8D). Moreover, we compared the potencies of muscarine, pilocarpine, carbachol and acetylcholine alone and in the presence of 1 µM isoprenaline in Table 1. There were no significant differences in potencies between drugs and the absence or presence of Iso (*p* < 0.05). However, it was not possible to calculate an EC50-value for muscarine alone (Table 1 and Fig. 2A and C).

**Table 1:** Comparison of potencies of muscarinic receptor agonists

Drug	Without Iso		With Iso	
	Log (EC_50_)	95% CI	Log (EC_50_)	95% CI
Muscarine	N.D		− 7.2	− 7.47 to − 6.92
Pilocarpine	− 6.13	− 6.88 to − 5.39	− 6.26	− 6.66 to − 5.85
Carbachol	− 6.96	− 7.81 to − 6.68	− 7.25	− 7.27 to − 6.66
Acetylcholine	− 6.18	− 6.93 to − 5.45	− 6.6	− 7.18 to − 6.03

Comparison of potencies of the drugs of interest alone or in the presence of 1 µM isoprenaline (Iso) on the force of contraction in HAP. EC_50_ values of muscarinic receptor agonists in the presence or absence of isoprenaline (Iso) in human atrial preparations. Data are given as decadic logarithm (EC_50_) together with the 95% confidence interval (CI). There were no significant differences in potencies between drugs (*p* < 0.05). Data were calculated from Figs. 2C, 6C, 7C, and 8C (therein also listed the numbers of experiments)

*N.D.* not determinable

**Fig. 9:**
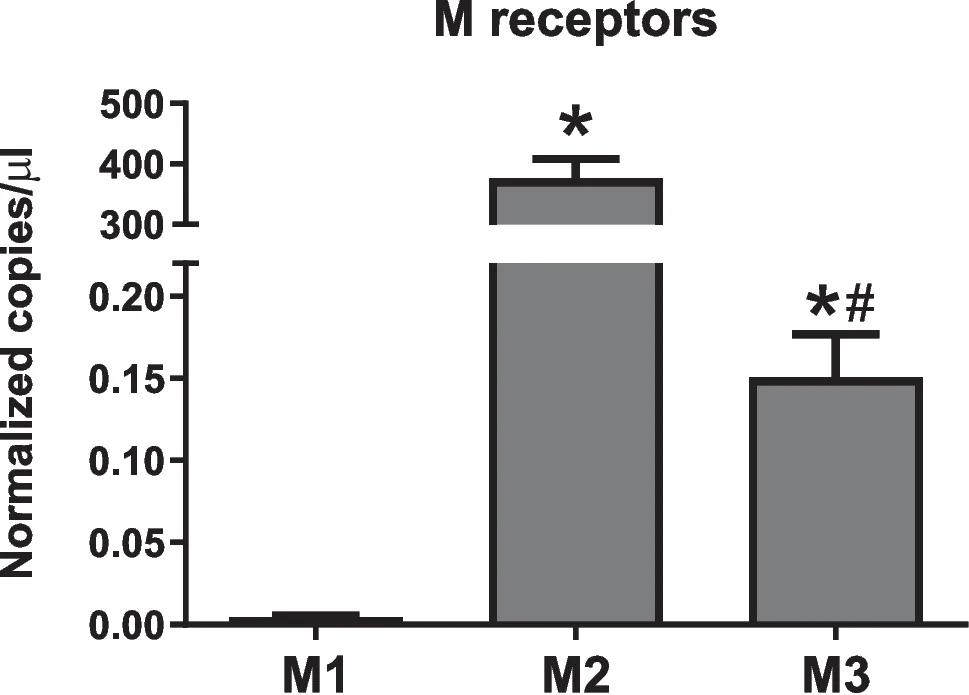
Muscarinic receptor expression: Expression levels of M_1_-muscarinic receptors (encoded by CHRM1), M_2_-muscarinic receptors (encoded by CHRM2) and M_3_-muscarinic receptors (encoded by CHRM3) receptors were quantified in human atrial tissue by dPCR analysis. The expression levels were normalized against the reference gene HPRT1. All experiments were performed in duplicate. Data are presented as mean ± SEM, *n* = 15; *p < 0.05 vs M_1_; ^#^*p* < 0.05 vs M_2_

Finally, the question arose, which muscarinic receptor is predominant in HAP. To this end, we measured in the HAP from 15 different patients the mRNA expression of the three muscarinic receptors of interest. The summarized results are plotted in Fig. 9. It turns out (please note the break in the ordinate) that the majority of the muscarinic receptors under our experimental conditions are the M_2_-muscarinic receptors (96.34%). A much smaller contribution comes from the M_3_-muscarinic receptor (3.57%) and very little contribution is noted for the M_1_-muscarinic receptor (0.09%). This relative contribution can also be found in the HAP from each individual patient (supplementary data 1). We had performed in nearly all samples (with the exception of two) in which we measured the mRNA (supplementary data 1) and performed contraction experiments with muscarine. We formed two groups: responders (initial decreases in force followed by an increase in force by muscarine, *n* = 4) and non-responders (only decreases in force by muscarine, *n* = 9). For these groups we listed the cp% for M1 in the mRNA (cf. supplementary data 1). When we performed a two-sided *t*-test on the two groups, we calculated a *p*-value of 0.0089, suggesting a significant difference between groups.

## Discussion

Muscarine may be regarded as the founding stone of modern pharmacology (Schmiedeberg and Koppe 1869). It was pivotal in defining the role of the sympathetic and parasympathetic nervous system in humans (Goodman and Gilman 2023). The main new finding in this report was that muscarine exerted a direct and an indirect negative inotropic effect in HAP. To study the indirect negative inotropic effects of muscarine we used a wide variety of cAMP-increasing agents or cAMP-mimicking (dibutyryl-cAMP) positive inotropic agents. A minor new finding was that pilocarpine was more effective to reduce FOC in the presence than in the absence of isoprenaline in HAP. A negative inotropic effect of carbachol or acetylcholine, either alone or in the presence of β-adrenergic stimulation in HAP, has been reported before and was studied for comparison with muscarine (e.g., from failing human hearts: Jacob et al. 1989; Böhm et al. 1994; Schwarz et al. 2023; from non-failing human hearts: Du et al. 1995).

There is consensus in the field that β-adrenoceptors increase cAMP levels in human atrial cardiomyocytes, augment the activity of the cAMP-dependent protein kinase and lead to the phosphorylation of, e.g., phospholamban and the activation of, e.g., L-type calcium ion channels. This pathway leads eventually to a positive inotropic and a lusitropic effect to, e.g., isoprenaline (Fig. 1).

It is clear that the effect of muscarine is M-cholinoceptor mediated because it is reversed by atropine. More specifically, the effects of muscarine are likely mediated by the M_2_-muscarinic receptor. Indeed, the effect of muscarine is reversed by AF-DX, a compound with some selectivity for M_2_-muscarinic receptors. Experiments in HAP by other groups in the past indicated that acetylcholine and/or carbachol, and, by extrapolation, muscarine act via the M_2_-muscarinic receptor (Du et al. 1995, Dhein et al. 2001). Other monoamines like serotonin and histamine exert, via their appropriate receptors (i.e., 5-HT_4_-serotonin receptors, H_2_-histamine receptors), a positive inotropic effect in the human heart more specifically in HAP. Like for isoprenaline, also these processes involve cAMP and the cAMP-dependent protein kinase (e.g., Gergs et al. 2009, 2017, 2019). The positive inotropic effects of serotonin and histamine in HAP were attenuated by muscarine. Even the positive inotropic effects via the glucagon-like-peptide-1 receptor in HAP (using 100 nM semaglutide as in our previous publication, Neumann et al. 2024) were attenuated by muscarine, indicating a more general mechanism than a mechanism involving only the β-adrenoceptors.

The question arose on the mechanism of the negative inotropic effect of muscarine. To this end, we applied drugs that increase cAMP in a receptor-independent way: we employed cilostamide because it inhibits degradation of cAMP in HAP. Interestingly, even in the presence of cilostamide, muscarine reduced the force of contraction. This indicates that muscarine can act without regard to how cAMP is generated (Fig. 1): muscarine reduced the force of contraction raised by many G-protein-coupled receptors (β-adrenoceptors, glucagon-like-peptide-1 receptors, H_2_-histamine receptor, 5-HT_4_-serotonin receptors and D_1_-dopamine receptors). Forskolin increases cAMP independent of any receptor stimulation. Also in the presence of forskolin, muscarine reduced the force of contraction. Moreover, we used a cell-membrane permeant derivative of cAMP, which is dibutyryl-cAMP (Rayo-Abella et al. 2023; Schwarz et al. 2023). Dibutyryl-cAMP raises the force of contraction because it mimics cAMP. But this positive inotropic effect of dibutyryl-cAMP is independent of an increased generation of cAMP by receptor stimulation or an impediment to cAMP degradation by a phosphodiesterase. Indeed, even in the presence of dibutyryl-cAMP, muscarine reduced the force of contraction. In the synopsis (Fig. 1), our findings suggest that other mechanism(s) could underlie, at least in part, the indirect negative inotropic effects of muscarine in HAP. We obtained similar results with carbachol in HAP (Schwarz et al. 2025). We speculate that muscarine may decrease the force of contraction in HAP, at least in part, by stimulation of other processes like activation of phosphatases (Fig. 1); in other words, muscarine may act independent from any stimulation of the cAMP-dependent protein kinase by reducing protein phosphorylation.

Muscarine can cause death due to hypotension and bradycardia or arrhythmias in general (Pauli and Foot 2005). Muscarine has no ester linkage and hence muscarine is not degraded and inactivated by a plasma choline esterase (Pauli and Foot 2005). Being polar, the uptake into the body through the gastrointestinal tract is thought to be poor. Consistent with that assumption, the toxicity in animals of peroral muscarine is at least tenfold lower than when muscarine is applied parenterally. However, transporters for the quaternary amine choline are now known which might also transport muscarine through cell membranes (Inazu 2014). This probably explains why muscarine even given per orally can cause an intoxication (Lurie et al. 2009).

The stimulatory effect of pilocarpine on muscarinic receptors is sometimes used for diagnostic purposes (sudomotoric function, Gerrett et al. 2021) but also to treat xerostomia, e.g., dry mouth after local radiation therapy. More recently, pilocarpine has been approved in eye drops to treat presbyopia and this beneficial effect is explained by miosis due to M_3_-cholinoceptor stimulation in the eye (Zhang et al. 2024).

The effect of pilocarpine alone on the force of contraction in the human atrium has apparently also not yet been reported. Only a negative inotropic effect of pilocarpine in the presence of β-adrenergic stimulation in HAP was reported previously (Delhaye et al. 1984). Pilocarpine was less potent and less effective than e.g., carbachol to reduce the force of contraction in the presence of isoprenaline in the human heart. A plausible explanation for this observation comes from previous binding studies where pilocarpine was a partial agonist at M_2_-muscarine receptors (Dunlap and Brown 1984). Others already reported that pilocarpine in the presence of isoprenaline reduced the force of contraction in HAP via M_2_-muscarine receptors (Delhaye et al. 1984). Likewise, pilocarpine was less potent than carbachol to reduce the force of contraction in rat left atrial preparations (Zanesco et al. 1999) and pilocarpine was less potent than acetylcholine to reduce beating rate in guinea pig right atrial preparations (Dorofeeva et al. 1992; Wang et al. 1999). Hence, the lack of a negative inotropic effect of pilocarpine in HAP might result from a stimulation of M_1_-muscarinic receptors which overcomes the negative inotropic effect of M_2_-muscarinic receptor stimulation (Du et al. 1995). In contrast, after isoprenaline, pilocarpine reduced the force of contraction which is probably mediated by M_2_-muscarinic receptors as already reported by Delhaye et al. 1984.

“Indirect” anti-β-adrenergic effects of carbachol are known to occur in the human atrium in isolated muscle strips from the human atrium (e.g., Genovese et al. 1988; Du et al. 1994). The negative inotropic effects of carbachol vanish in the atrium from M_2_-muscarinic receptor-knock-out mice and thus are M_2_-muscarinic receptor mediated at least in mice (review, Dhein et al. 2001). Similar findings are seen in human atrial samples, here studies with receptor specific antagonists indicate that the negative inotropic effects of acetylcholine are M_2_-mediated (Du et al. 1995; Dhein et al. 2001). Hence, we suggest that muscarine also reduces the force of contraction via M_2_-muscarinic receptors in HAP.

Positive inotropic effects of M-cholinoceptor agonists have been observed for decades but never gained much attention (e.g., Jacob et al. 1989). Some studies indicate that the positive inotropic effect of M-cholinoceptor agonists always occurs after the negative inotropic effect. The positive inotropic effect of M-cholinoceptor agonists was mediated by M_2_-cholinoceptors and M_1_-muscarinic receptors in HAP as judged from contractile studies with several antagonists (Du et al. 1995).

We have also provided measurements of the expression of these three muscarinic receptors in human atrial samples. In HAP, we mainly found M_2_-muscarinic receptors, fewer M_3_-muscarinic receptors, and a rather small expression of M_1_-muscarinic receptors. Others before have measured the expression of the mRNA in HAP. They detected the mRNA for M_1_-, M_2_-, M_3_-, M_4_-, and M_5_-muscarinic receptors in the human atrium (Wang et al. 2001; Perez et al. 2006; Hejman et al. 2018). In contrast, Oberhauser et al. (2001) also detected M_2_-muscarinic and M_3_-muscarinic, but they reported that M_1_-muscarinic receptors were absent in the human atrium and did not try to measure M_4_-muscarinic receptors and M_5_-muscarinic receptors. However, they (Oberhauser et al. 2001; Wang et al. 2001; Perez et al. 2006) did not quantify their mRNA expression data or only calculated the expression between diseased (chronic atrial fibrillation) and normal atrial preparations (Heijman et al. 2018). Our quantifications in HAP are plausible because they are in agreement with previous quantitative reports of the mRNA in rat heart; also, about 94% were identified as M_2_-muscarinic, 0.8% as M_1_-muscarinic, and 1.1% as M_3_-muscarinic receptors (Krejci and Tucek 2002; Kitazawa et al. 2009). Similarly, in the mouse heart, 99.87% were identified as M_2_-muscarinic, 0.3% as M_1_-muscarinic, and 0.05% as M_3_-muscarinic receptors (Kitazawa et al. 2009). While few M_1_-muscarinic receptors are present in the human atrium, they increased potassium channel activities in the human atrium (Heijman et al. 2018) and may underlie positive inotropic effects of acetylcholine in the human atrium (Du et al. 1995). Interestingly, we have found some evidence that increased levels of the mRNA of the M_1_-muscarinic receptor expression might explain the positive inotropic effects of muscarine in HAP which we noted in some patients.

### Clinical relevance for drug therapy

Carbachol, pilocarpine and muscarine have in common that they are not degraded in contrast to acetylcholine by a choline esterase (Goodman and Gilman 2023). Carbachol is still used in anesthesiology and after bowel surgery to re-start the gut contraction (Goodman and Gilman 2023). Parenterally, applied acetylcholine is sometimes used in cardiology for diagnostic purposes (e.g., detecting a sick sinus syndrome or testing the function of the endothelium) (Goodman and Gilman 2023). Intravenous pilocarpine decreases blood pressure in human volunteers (Tanzer et al. 1995). Pilocarpine eye drops have been used since 1877 to treat glaucoma by inducing miosis (Weber 1877). Pilocarpine from local application in eye drops that contain pilocarpine when they are absorbed via the nasolacrimal system or the eye conjunctiva can probably affect cardiovascular function. Indeed, in clinical studies pilocarpine eye drops reduced systolic blood pressure (Yamashita et al. 2010; Seibold et al. 2018). This could result from direct vasodilatory effects of pilocarpine on the resistance vessels or theoretically by stimulation of cardiac M_2_-muscarinic receptors in the left ventricle, which at present is speculative. In mice and men, M_2_-cholinoceptor receptors are known to mediate negative chronotropic effects or atrioventricular block (Littmann et al. 1987; Van Gelderen et al. 1996). Like carbachol, muscarine is predicted to reduce the force of contraction and the beating rate when applied e.g., in an intoxication in humans. Such intoxications with muscarine-containing mushrooms have been repeatedly described from intoxication centers in several countries (Lurie et al. 2009). These authors report arrhythmias and reduced blood pressure, which is consistent with our present data (Lurie et al. 2009; Flament et al. 2020; Graeme 2014; Patocka et al. 2021; Balice et al. 2024).

### Limitations

We did not study the biochemical alterations in protein phosphorylation in the human atrium due to muscarine. We predict that isoprenaline-stimulated phosphorylation of phospholamban would be decreased by muscarine. In a confirmatory fashion, one should measure with fluorescent dyes the cAMP levels and Ca^2+^-transients in primary cultures of electrically stimulated human atrial cardiomyocytes to ascertain that after application of muscarine, cAMP levels remain unchanged in all compartments of the cardiomyocyte and Ca^2+^-transients as well as the phosphorylation state of phospholamban would decline, which would be our prediction. Moreover, we have not yet directly studied how muscarine might activate cardiac phosphatases, which is our preferred speculation for the mechanism of muscarine in the heart.

In summary, we noted that muscarine alone and muscarine in the presence of drugs that activate cAMP-dependent protein kinases had a negative inotropic effect in the isolated human atrium.

## Supplementary Information

Below is the link to the electronic supplementary material.

ESM 1 (PDF 549 KB)

## Data Availability

All source data for this work (or generated in this study) are available upon reasonable request.
