# Supplementary material for: On indirect negative inotropic effects of muscarine in the isolated human atrium
Source: Naunyn Schmiedebergs Arch Pharmacol. 2026 Jul 14;399(13):21021–41. doi: 10.1007/s00210-026-05666-2 (PMC13695641; doi:10.1007/s00210-026-05666-2)
Supplement: Supplementary file 1 [file 210_2026_5666_MOESM1_ESM.pdf]

Supplementary table 1

Digital PCR (dPCR) data are presented as absolute mRNA copy numbers per microliter (cp/µL) for each individual sample, along with the relative contribution of each muscarinic receptor subtype (M1, M2, M3) to the total M receptor transcript pool (expressed as a percentage of 100%) in 15 different patients (HAP#).

Sample	***M1 receptor***		***M2 receptor***		***M3 receptor***	
	cp/ µl	***cp%***	***cp/ µl***	***cp%***	***cp/ µl***	***cp%***
HAP1	0.92	0.18	491.36	97.76	10.35	2.06
HAP2	0.3	0.06	448.32	97.1	13.08	2.83
HAP3	0.1	0.03	306.8	94.8	16.71	5.16
HAP4	0.52	0.11	465.7	97.31	12.36	2.58
HAP5	0.41	0.09	431.9	99.15	3.3	0.76
HAP6	0.3	0.08	379.3	96.74	12.47	3.18
HAP7	0.33	0.13	247.17	93.46	16.96	6.41
HAP8	0.22	0.06	367.02	97.79	8.07	2.15
HAP9	0.46	0.21	204.61	93.62	13.48	6.17
HAP10	0.26	0.09	276.29	97.46	6.93	2.44
HAP11	0.26	0.05	533.9	98.72	6.65	1.23
HAP12	0.39	0.18	191.3	89.94	21.02	9.88
HAP13	0.26	0.04	623.8	96.32	23.55	3.64
HAP14	0.3	0.1	294.37	98.07	5.51	1.84
HAP15	0.03	0.01	382.06	96.84	12.44	3.15
**average**		**0.09**		**96.34**		**3.57**
